# Multiomics Reveals Nonphagocytosable Microplastics Induce Colon Inflammatory Injury via Bile Acid-Gut Microbiota Interactions and Barrier Dysfunction

**DOI:** 10.1021/acsami.5c07250

**Published:** 2025-07-19

**Authors:** Junjie Chen, Yixian Cheng, Rui Fu, Xinyu Chen, Peng Zhang, Yixiao Lu, Bingsheng Liu, Peng Chen, Jiahao Wang, Haikun Cao, Jinghua Gu, Haosong Chen, Zilong Jiang, Ting Li, Jiawei Zhang, Bo Chen, Guodong Cao

**Affiliations:** † Department of General Surgery, 36639 The First Affiliated Hospital of Anhui Medical University, Hefei, Anhui 230022, China; ‡ Department of General Surgery, The Second Affiliated Hospital of Anhui Medical University, Hefei, Anhui 230601, China; § Department of Surgical Oncology, 74540 The First Affiliated Hospital of Bengbu Medical University, Bengbu, Anhui 233004, China; ∥ Department of Medical Oncology, The First Affiliated Hospital of Anhui Medical University, Hefei, Anhui 230022, China

**Keywords:** microplastics, colon inflammation, barrier dysfunction, bile acid metabolism, gut microbiota

## Abstract

Microplastics (MPs), as emerging global environmental pollutants, exhibit intestinal toxicity mechanisms that are closely associated with the particle size. Nonphagocytosable MPs (NPMs), though incapable of being internalized by intestinal epithelial cells, still provoke colonic inflammatory damage. However, the exact mechanisms remain elusive. This study established a BALB/c mouse model subjected to long-term oral exposure to 10 μm polystyrene MPs (PS MPs) to comprehensively explore how NPMs induce colonic inflammation and injury. The results demonstrate that prolonged PS MPs exposure disrupts the colonic redox balance, leading to oxidative stress. Simultaneously, it disturbs intestinal immune homeostasis by elevating the Th17/Treg cell ratio and upregulating pro-inflammatory cytokines. Additionally, PS MPs notably compromise intestinal mechanical barrier function, diminishing mucin secretion and downregulating tight junction protein expression. Multiomics analysis further uncovered that PS MPs induce bile acid (BA) metabolic dysregulation by interfering with liver function and gut microbiota, causing a marked accumulation of total bile acids in the colon, especially conjugated BAs. Both in vitro and in vivo experiments confirmed that specific concentrations of taurochenodeoxycholic acid (TCDCA) activate the reactive oxygen species-mitochondrial pathway, triggering apoptosis in colonic epithelial cells and exacerbating PS MPs-induced colonic inflammatory injury. This study provides the first evidence of a cross-organ regulatory mechanism in which NPMs mediate intestinal toxicity via the “liver-BA-gut axis,” offering novel theoretical insights for assessing the intestinal toxicity of MPs.

## Graphical abstract

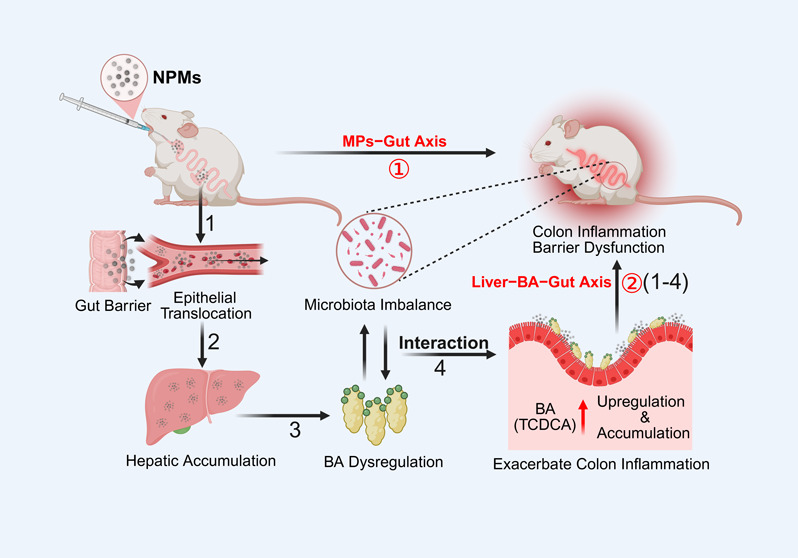

## 1 Introduction

Plastic pollution has emerged as a pervasive and increasingly severe environmental challenge on a global scale. Over 9.2 billion tons of plastic have been produced worldwide, but more than 90% of plastic waste remains unrecycled.^1^ Influenced by various physical and chemical processes in the environment, discarded plastics degrade into microplastics (MPs, <5 mm) and even smaller nanoplastics (NPs, <1 μm).^2,3^ As a newly recognized global pollutant, MPs are widely dispersed across aquatic systems, soil, and the atmosphere, entering the human body through inhalation, dietary intake, water consumption, and skin contact.^4^ Humans are continuously exposed to low concentrations of MPs throughout their lifespan, with an estimated average weekly intake of 0.1–5 g, potentially posing significant health risks.^5^ MPs are present in various human organs, tissues, body fluids, and excretions, prompting growing concerns about their adverse effects on human health.^4^ Oral ingestion remains the primary route of human exposure to MPs, where they not only affect the intestines but may also cross the intestinal barrier, enter the bloodstream, and spread throughout the body.^6^ Consequently, the intestines are critical target organs for MPs, serving as a focal point for their toxic effects. Although numerous studies have demonstrated that MPs can cause intestinal inflammation and disrupt the intestinal barrier, the specific mechanisms driving their intestinal toxicity remain to be fully elucidated.^7–9^

The biological toxicity of MPs is influenced by various factors, including size, morphology, composition, degree of aging, and surface properties, with particle size being the most critical determinant of biodistribution and the extent of toxicity.^10–12^ Particle size significantly impacts the biodistribution of MPs within the body, influencing their localization in different tissues and the degree of accumulation in those tissues.^13^ Thus, MPs of varying sizes can exhibit differences in the type, degree, and mechanisms of toxicity within the same cells or tissues/organs. For example, Wang et al. demonstrated that at identical exposure concentrations, PS MPs of different sizes could induce damage to the bladder epithelium in mice, with distinct injury types and mechanisms.^14^ PS MPs in the 1–10 μm range triggered significant necroptosis through oxidative stress, while PS MPs of 50–100 μm primarily induced a stronger inflammatory response via the p-NFκB p65 pathway. Similarly, Wen et al. showed that MPs of different sizes could produce similar effects through varying molecular mechanisms.^15^ Exposure of C57BL/6 male mice to 80 nm and 5 μm PS MPs resulted in reduced spermatocyte counts in the seminiferous tubules, impairing spermatogenesis. The 80 nm PS MPs reduced spermatocytes by altering retinoic acid metabolism, whereas the 5 μm PS MPs did so by disrupting thyroid hormone metabolism. At the cellular level, Wang et al. found that larger PS particles (500 and 1000 nm) typically caused hepatocyte death by disrupting the cell membrane, while smaller particles (20 nm) were more likely to penetrate cells, inducing severe oxidative stress and leading to hepatocyte death.^16^ Similarly, in the intestines, MPs’ toxicity is closely linked to particle size, with distinct mechanisms of intestinal inflammation, barrier disruption, and other injuries based on particle size. It is generally accepted that MPs’ biological toxicity is size-dependent, with smaller particles demonstrating greater toxicity. Smaller MPs are more readily phagocytosed and can penetrate intestinal epithelial cells, making their hazards to the intestines more evident.^17^ However, some researchers argue that no direct correlation exists between MPs’ size and toxicity, asserting that toxicity depends not only on the adsorption capacity of the target organ but also on the presence and stimulation pathways of MPs within that organ.^18,19^ In our preliminary animal experiments, 10 μm PS MPs were specifically studied and found to be nonphagocytosable by intestinal epithelial cells. Despite this, these nonphagocytosable microplastics (NPMs) still induced significant intestinal inflammatory damage, and the underlying mechanisms remain unclear, warranting further investigation.

As the primary organ responsible for metabolism and detoxification, the liver is the first critical site of contact for MPs after they pass through the intestines. Upon oral ingestion, some MPs can cross the intestinal barrier, enter the bloodstream, and reach the liver via the enterohepatic circulation, where they accumulate and potentially cause long-term adverse effects. Numerous animal studies have demonstrated that chronic exposure to MPs can result in liver inflammation, hepatic fibrosis, and disruptions in glucose and lipid metabolism.^20–22^ Furthermore, once MPs enter the liver, they can interfere with the bile acid (BA) metabolism, disrupting the production and secretion of BAs. PS MPs significantly elevate total BA (TBA) levels in the liver.^23,24^ However, the impact and underlying mechanisms of these elevated BAs on intestinal inflammation and the intestinal barrier remain unclear. The gut microbiota play a critical role in maintaining intestinal homeostasis by modulating BA metabolism, and bile acids in turn influence the composition and function of the microbiota. Excessive bile acids entering the intestine can disrupt the balance of the gut microbiota, compromise the integrity of the intestinal barrier, and exacerbate intestinal inflammation.^25^ Additionally, research on the liver-BA-gut axis in the context of MPs-induced intestinal inflammatory injury is still limited.

In this study, a 6-week gavage model was established using 10 μm PS MPs, a type of NPMs, in male BALB/c mice to explore the mechanisms by which NPMs induce colon inflammatory injury. Through multiomics analysis, BA metabolism dysregulation and gut microbiota imbalance in fecal samples were examined. Finally, how BA metabolism disruption exacerbates colon inflammatory injury in mice was investigated. These findings offer novel insights into the mechanisms by which oral exposure to NPMs induces colon inflammatory damage.

## 2 Materials and Methods

### 2.1 Materials

PS MPs are typical microplastics widely present in the environment and commonly used for toxicity studies.^26^ This study utilized two types of PS MPs (Zhichuan, Jiangsu, China). Conventional PS MPs, with a particle size of 10 μm, were used for toxicological research in animal and cell experiments. Additionally, fluorescent PS MPs (excitation wavelength: 520 nm; emission wavelength: 580 nm) with particle sizes of 100 nm, 1 μm, and 10 μm were employed to visualize ingestion, accumulation, and distribution in vivo and in vitro. The morphology and particle size of the PS MPs were confirmed using scanning electron microscopy (SEM, Sigma 300, ZEISS, Germany) and a laser particle size analyzer (Mastersizer 2000, Malvern, UK). The chemical composition of the PS MPs was verified through Fourier-transform infrared spectroscopy (FTIR; IRTracer 100, Shimadzu, Japan). Taurochenodeoxycholic acid (TCDCA) was obtained from Aladdin (CAS#: 516-35-8, Shanghai, China). The cell counting kit-8 (CCK-8), calcein/propidium iodide (PI) Live/Dead viability/cytotoxicity assay kit, reactive oxygen species (ROS) assay kit, and mitochondrial membrane potential assay kit with JC-1 were provided by Beyotime Biotechnology, Shanghai, China. The Annexin V-FITC/PI apoptosis detection kit was supplied by Jiangsu KeyGEN Biotechnology, China.

### 2.2 Animal Culture

SPF BALB/c mice (4 weeks old, male) were obtained from the Anhui Experimental Animal Center (Hefei, Anhui, China). The mice were housed at 22–26 °C with controlled humidity and a 12 h light/dark cycle, with 1 week of acclimatization. All animal experiments utilized 10 μm PS MPs, diluted in PBS to a final concentration of 10 mg/mL. All experimental protocols were approved by the Animal Care and Use Committee of Anhui Medical University (ethics approval number: LLSC20242418).

### 2.3 Cell Culture

The human normal colonic epithelial cell line (NCM460) was sourced from Warner Bio (Wuhan, China). NCM460 cells were cultured in RPMI1640 medium (Gibco, Waltham, MA, USA) supplemented with 10% fetal bovine serum (FBS; Gibco), 100 IU/mL penicillin, and 100 μg/mL streptomycin at 37 °C with 5% CO_2_. Once the cell density exceeded 90%, they were seeded into six-well plates, 24-well plates, or 96-well plates for subsequent experiments.

### 2.4 In Vivo Live Imaging of Single High-Dose Oral Exposure of Red Fluorescent PS MPs in Mice

Six healthy mice were randomly selected and fasted for 8 h. Fluorescent PS MPs at a concentration of 10 mg/mL were orally administered at 0, 2, 4, 8, and 12 h, with a gavage volume of 200 μL per mouse. The control group received an equivalent volume of PBS. Mice were anesthetized with pentobarbital sodium administered intraperitoneally. Using Small Animal In Vivo Optical Imaging (IVIS Luminz iii, PerkinElmer, USA), images were captured at each time point to monitor the ingestion, digestion, and accumulation of fluorescent PS MPs throughout the digestive tract, from the oral cavity to the anus, as well as their distribution in various organs.

### 2.5 Accumulation of Fluorescent PS MPs in Various Organs of Mice following Long-Term Oral Exposure

Five healthy mice were randomly selected and orally administered fluorescent PS MPs at a concentration of 10 mg/mL, with a daily dosage of 1 mg per mouse for 6 weeks. After the exposure period, the mice were euthanized, and tissue samples, including the heart, lungs, liver, spleen, kidneys, stomach, and colon, were collected. The tissues were fixed in 4% paraformaldehyde, embedded in paraffin, and sectioned to a thickness of 4 μm. Hematoxylin and eosin (H&E) staining was performed for observation. To detect the presence of fluorescent PS MPs in tissue sections, a bright-field image was first obtained using a Leica upright fluorescence microscope, followed by fluorescence imaging in the appropriate channel. The two sets of images were then merged for analysis.

### 2.6 Construction of the Mouse Colitis Model was Induced by Oral Exposure to PS MPs

After 1 week of acclimatization, 10 healthy mice were randomly selected and weighed. They were subsequently divided into two groups: the control group (NC) and the model group (MP), each consisting of 5 mice. During gavage, the body weight of the mice was measured every other day. The MP group received a 10 mg/mL concentration of PS MPs at a dosage of 1 mg/day per mouse,^27^ while the control group was administered an equivalent volume of PBS. Gavage continued for 6 weeks. Body weights of both groups were measured before the final gavage, and mice were fasted for 8 h after the final gavage. Euthanasia was carried out by cervical dislocation following intraperitoneal anesthesia with pentobarbital sodium. Tissues, including serum, feces, heart, lungs, liver, spleen, kidneys, stomach, cecum, and colon, were collected.

### 2.7 Uptake of Fluorescently Labeled PS MPs of Different Sizes by Normal Colonic Epithelial Cells

An appropriate number of NCM460 cells were seeded in a 24-well plate and cultured for 24 h until stable growth and proliferation were achieved. Fluorescent PS MPs of varying sizes (100 nm, 1 μm, 10 μm) were then coincubated with the cells at a concentration of 1 mg/mL for another 24 h. After coincubation, the wells were washed several times with PBS to remove uninternalized MPs. Bright-field, red fluorescence, and DAPI fluorescence images were captured using a live cell workstation (Celldiscoverer 7, Zeiss, Germany). The images were exported and merged using the ZEN software provided by Zeiss as necessary.

### 2.8 Tissue Histopathology Staining

Histopathological examinations were performed to assess inflammation in the colon and liver, as well as colonic mucus secretion. Colon and liver tissue samples were fixed in 4% paraformaldehyde, dehydrated, paraffin-embedded, and sectioned into 4 μm slices. The sections were stained with H&E and alcian blue periodic acid-Schiff (AB-PAS) reagents from Solarbio (Beijing, China). Tissue sections were observed and images were captured using a Leica upright microscope (DM6B, Leica, Germany).

### 2.9 Detection of Inflammatory Factors and TBAs by ELISA

Enzyme-linked immunosorbent assay (ELISA) kits were used to determine the expression levels of inflammatory factors in colon tissue and TBA levels in the liver, colon, and feces. The ELISA kits, provided by Meibiao Biotechnology (Jiangsu, China), included assays for mouse tumor necrosis factor-α (TNF-α), interleukin-1 β (IL-1β), interleukin-6 (IL-6), and interleukin-10 (IL-10). Mouse TBA ELISA kits were obtained from Zhongke Quality Inspection Biotechnology (Beijing, China). After supernatants were extracted from colon, liver, and fecal samples, procedures were strictly followed according to the manufacturer’s protocols for the respective commercial ELISA kits. Optical density (OD) values were measured at a wavelength of 450 nm by using a microplate reader (BioTek, USA). Data analysis involved constructing standard curves, and the expression levels of inflammatory factors and TBA were calculated based on these curves.

### 2.10 Detection of Oxidative Stress Biomarkers

For the detection of oxidative stress biomarkers in the colon and liver, mouse colon and liver tissue homogenates were prepared according to the kit manufacturer’s instructions. The levels of reduced glutathione (GSH) and oxidized glutathione (GSSG) were assessed by using a colorimetric assay (GSH and GSSG assay kit, S0053, Beyotime, Shanghai, China). Glutathione peroxidase (GSH-Px) content was determined using the NADPH method (total glutathione peroxidase assay kit, S0058, Beyotime). Superoxide dismutase (SOD) content was measured using the WST-8 method (total SOD activity assay kit S0101S, Beyotime). Malondialdehyde (MDA) levels were determined using an MDA content assay kit (BC0025, Solarbio), and catalase (CAT) activity was measured using a catalase activity assay kit (BC0205, Solarbio). Protein concentrations were determined using a BCA protein assay kit (P0012, Beyotime).

### 2.11 Assessment of Th17/Treg Balance

Splenic lymphocytes were isolated from murine specimens, followed by erythrocyte depletion using hypotonic lysis. After centrifugation and washing with PBS, cellular suspensions were preincubated with an Fc receptor-blocking agent to prevent nonspecific antibody interactions. Surface epitopes were labeled for 30 min at 37 °C using the following fluorophore-conjugated monoclonal antibodies: PB450-anti-CD45, FITC-anti-CD4, APC-anti-CD8, PerCP/Cy5.5-anti-CD3, and PE-anti-CD25. Intracellular staining was performed after fixation and permeabilization with APC-conjugated Foxp3 and PE/Cy7-tagged IL-17A antibodies under identical thermal conditions. Processed samples were resuspended in a stabilization buffer and analyzed by using flow cytometry (Beckman Coulter CytoFLEX platform). Quantitative data interpretation was performed using FlowJo software (version 10.8.1, Tree Star Inc.).

### 2.12 Tissue Immunofluorescence

Paraffin-embedded mouse colon tissue blocks were sectioned to a thickness of 3 μm and dewaxed in water. Endogenous peroxidase activity was blocked using an endogenous peroxidase blocking solution (AR1108, Boster, Wuhan, China) for 10 min at room temperature, followed by a wash with PBS. Antigen retrieval was performed using a microwave, and the tissue was then blocked with 5% BSA (BS114-5g, Biosharp, Hefei, Anhui, China) at room temperature for 2 h. Primary antibodies, including ZO-1 (1:200 dilution, 21773-1-AP, Proteintech, Wuhan, China), Occludin (1:200 dilution, 27260-1-AP, Proteintech), Claudin 1 (1:200 dilution, 13050-1-AP, Proteintech), and MUC1 (1:100 dilution, ET1611-14, Huabio, Hangzhou, China), were incubated overnight at 4 °C. The sections were then brought to room temperature, washed with PBS to remove unbound primary antibodies, and dried by gently wiping away excess water. Subsequently, the sections were incubated in the dark with FITC-labeled secondary antibodies (BL033A, Biosharp) at room temperature for 2 h. After incubation, the sections were washed with PBS on a shaker and mounted using an antifade mounting medium containing DAPI (P0131, Beyotime). Observations and imaging were performed with a Leica upright microscope.

### 2.13 Detection of Bile Acids

Feces (20 mg) were ground and extracted with 200 μL of methanol/acetonitrile (v/v = 2:8), then stored at −20 °C for 10 min for quantification. The mixture was then centrifuged for 10 min at 12,000 rpm and 4 °C to obtain the supernatant. The supernatant was analyzed using an liquid chromatography-electrospray ionization-tandem mass spectrometry (LC-ESI-MS/MS) system equipped with a Waters ACQUITY UPLC HSS T3 C18 column (100 × 2.1 mm i.d., 1.8 μm), consisting of UHPLC (ExionLC AD) and MS (Applied Biosystems 6500 Triple Quadrupole). Mobile phase A consisted of water with 0.01% acetic acid and 5 mmol/L ammonium acetate, while mobile phase B consisted of acetonitrile with 0.01% acetic acid. The column temperature was set to 40 °C, and the injection volume was 3 μL. Detection was carried out using the QTRAP 6500+ LC–MS/MS mass spectrometer system (SCIEX, USA), equipped with an ESI Turbo ion–spray interface, operating in negative ion mode, and controlled by Analyst 1.6.3 software (Sciex). The ESI source parameters were as follows: ion source, ESI; source temperature, 550 °C; ion spray voltage (IS), −4500 V; curtain gas (CUR), 35 psi.

### 2.14 16S rRNA Sequencing of Fecal Samples

Sequencing was conducted by MetWare (http://www.metware.cn/) using the NovaSeq 6000 platform (Illumina, USA). Following the acquisition of raw sequencing data, filtering and merging were performed with Fastp (v0.22.0), FLASH (v1.2.11), and Vsearch (v2.22.1) to generate effective tags. Operational taxonomic unit (OTU) clustering and amplicon sequence variant (ASV) denoising were carried out based on these effective tags. Taxonomic annotation and multiple sequence alignment analyses were then performed on the sequences of the OTU/ASV, providing species identification and abundance distribution profiles. Differences in the community structure across various samples or groups were explored. The data were subsequently normalized, followed by analyses such as alpha diversity, beta diversity, differential species analysis with significance testing, network analysis, and functional prediction based on the normalized data.

### 2.15 Cell Viability Assay

For cell viability assays, NCM460 cells were seeded in a 96-well plate. After 24 h, the culture medium was removed, and various concentrations of PS MPs or TCDCA, diluted in RPMI1640 medium, were added. After 24 h of incubation, CCK-8 reagent was added, followed by a 2 h incubation. The OD values at 450 nm were measured by using a microplate reader, and cell viability was calculated.

### 2.16 Cell Live/Dead Assay

NCM460 cells were also seeded into a 24-well plate and treated with specified concentrations of PS MPs or TCDCA for a designated period. Calcein AM/PI detection working solution was prepared according to the manufacturer’s instructions. After the culture medium was removed, cells were washed once with PBS, and residual liquid was removed. Then, 250 μL of the Calcein AM/PI working solution was added to each well, and the plate was incubated at 37 °C in the dark for 30 min. Following incubation, staining was observed under a fluorescence microscope and images were captured. Calcein AM emits green fluorescence (Ex/Em = 494/517 nm), while PI emits red fluorescence (Ex/Em = 535/617 nm).

### 2.17 Cell ROS Assay

For ROS detection, DCFH-DA was diluted in a serum-free culture medium at a 1:1000 ratio to achieve a final concentration of 10 μM. After removing the culture medium, an appropriate volume of diluted DCFH-DA was added to cover the cells, which were then incubated at 37 °C for 20 min. The cells were washed three times with a serum-free medium to remove any unincorporated DCFH-DA. Rosup was added to the positive control well. Finally, the cells were observed, and images were captured using a live-cell workstation at Ex/Em = 488/525 nm.

### 2.18 Mitochondrial Membrane Potential Assay

Following the manufacturer’s instructions, the JC-1 staining working solution was prepared. CCCP was diluted to 10 μM and used to treat the cells for 20 min as a positive control. The culture medium was removed from the 24-well plate, and the cells were washed once with PBS. Next, 1 mL of complete culture medium was added, followed by 1 mL of JC-1 staining working solution, which was mixed gently and thoroughly. The plate was incubated at 37 °C in a cell culture incubator for 20 min. During incubation, a 1× JC-1 staining buffer was prepared and kept on ice. After incubation, the supernatant was removed and the cells were washed twice with 1× JC-1 staining buffer. Finally, 1 mL of complete culture medium was added, and the cells were observed and imaged using a live-cell workstation. JC-1 aggregates emit red fluorescence (Ex/Em = 525/590 nm), while JC-1 monomers emit green fluorescence (Ex/Em = 490/530 nm).

### 2.19 Cell Apoptosis Detection

For apoptosis detection, cells from each group were first collected after trypsin digestion without EDTA. The cells were washed twice with PBS, centrifuged at 800 rpm for 5 min, and the supernatant was discarded. Then, 500 μL of binding buffer from the kit was added to the cells, mixed gently to form a single-cell suspension, and transferred to flow cytometry tubes. Next, 5 μL of Annexin V-FITC was added to each tube and mixed thoroughly, followed by the addition of 5 μL of PI to each tube and gently mixed again. The tubes were incubated in the dark at room temperature for 10 min. Within 1 h, flow cytometry was performed to observe and detect the cells. The green fluorescence of Annexin V-FITC (Ex/Em = 488/530 nm) was detected through the FITC channel (FL1), and the red fluorescence of PI (Ex/Em = 488/630 nm) was detected through the PI channel (FL3).

### 2.20 Western Blotting

For protein extraction, cells were lysed on ice using cell lysis buffer (P0013, Beyotime) containing the protease inhibitor PMSF (ST506, Beyotime, China). The protein concentration was determined using a BCA protein assay kit. Proteins were separated by 10% SDS-PAGE and transferred onto a PVDF membrane. The membrane was blocked in 5% skim milk for 1 h and then incubated overnight at 4 °C with appropriately diluted primary antibodies (Bax, Bcl-2, Caspase-3, Caspase-9, PARP, GAPDH). The next day, after washing three times with TBST, the membrane was incubated with corresponding secondary antibodies for 1 h. The membrane was washed three times with TBST, stained with enhanced chemiluminescence (ECL) reagents (Affinity, China), and imaged and analyzed using a chemiluminescence imaging system (Tanon, Shanghai, China).

### 2.21 In Vivo Validation of TCDCA Exacerbating Colitis

All mice were acclimated for 1 week and weighed before being randomly assigned to four groups: the control group (NC), the experimental group (MP), the MP + ABX group, and the MP + TCDCA group, with five mice per group. PS MPs were administered via gavage to all experimental groups at a concentration of 10 mg/mL, with a dosage of 1 mg per mouse per day, while the control group received an equivalent volume of PBS. Gavage was performed continuously for 6 weeks. In the MP + ABX group, an antibiotic mixture (ABX) was provided via drinking water, consisting of 0.5 g/L vancomycin (CAS#: 1404-93-9, Aladdin), 1 g/L neomycin sulfate (CAS#: 1405-10-3, Aladdin), 1 g/L metronidazole (CAS#: 443-48-1, Aladdin), and 1 g/L ampicillin (CAS#: 69-52-3, Aladdin). The solution was replaced every 3 days for 6 weeks to deplete the intestinal microbiota. From the fifth week onward, mice in the MP + TCDCA group were orally administered with TCDCA dissolved in PBS at a dose of 200 mg/kg^28^ for 2 weeks. Prior to the final gavage, all mice were weighed, fasted for 8 h, and euthanized via cervical dislocation under pentobarbital sodium anesthesia. Samples including serum, feces, heart, lungs, liver, spleen, kidneys, stomach, cecum, and colon tissues were collected.

### 2.22 Statistical Analysis

Data are presented as mean ± SEM. Statistical analyses were performed by using GraphPad Prism 10.1 software. One-way ANOVA followed by post hoc Tukey tests were used for statistical comparisons between groups. A *P*-value of <0.05 was considered statistically significant.

## 3 Results

### 3.1 Characterization of PS MPs

SEM was employed to capture the overall morphology of the PS MPs samples. Both low- and high-magnification images revealed that PS MPs of three different diameters displayed spherical structures with uniform sizes of 100 nm, 1 μm, and 10 μm, indicating excellent monodispersity (Figure 1A). Subsequently, a laser particle size analyzer was used to measure the size and distribution of the PS MPs, confirming that the average sizes were centered around 100 nm, 1 μm, and 10 μm, consistent with the SEM observations (Figure 1B). FTIR spectroscopy was then applied to analyze the chemical composition of the samples, and the characteristic absorption peaks of polystyrene were observed, confirming the material as polystyrene (Figure 1C).

**Figure 1:**
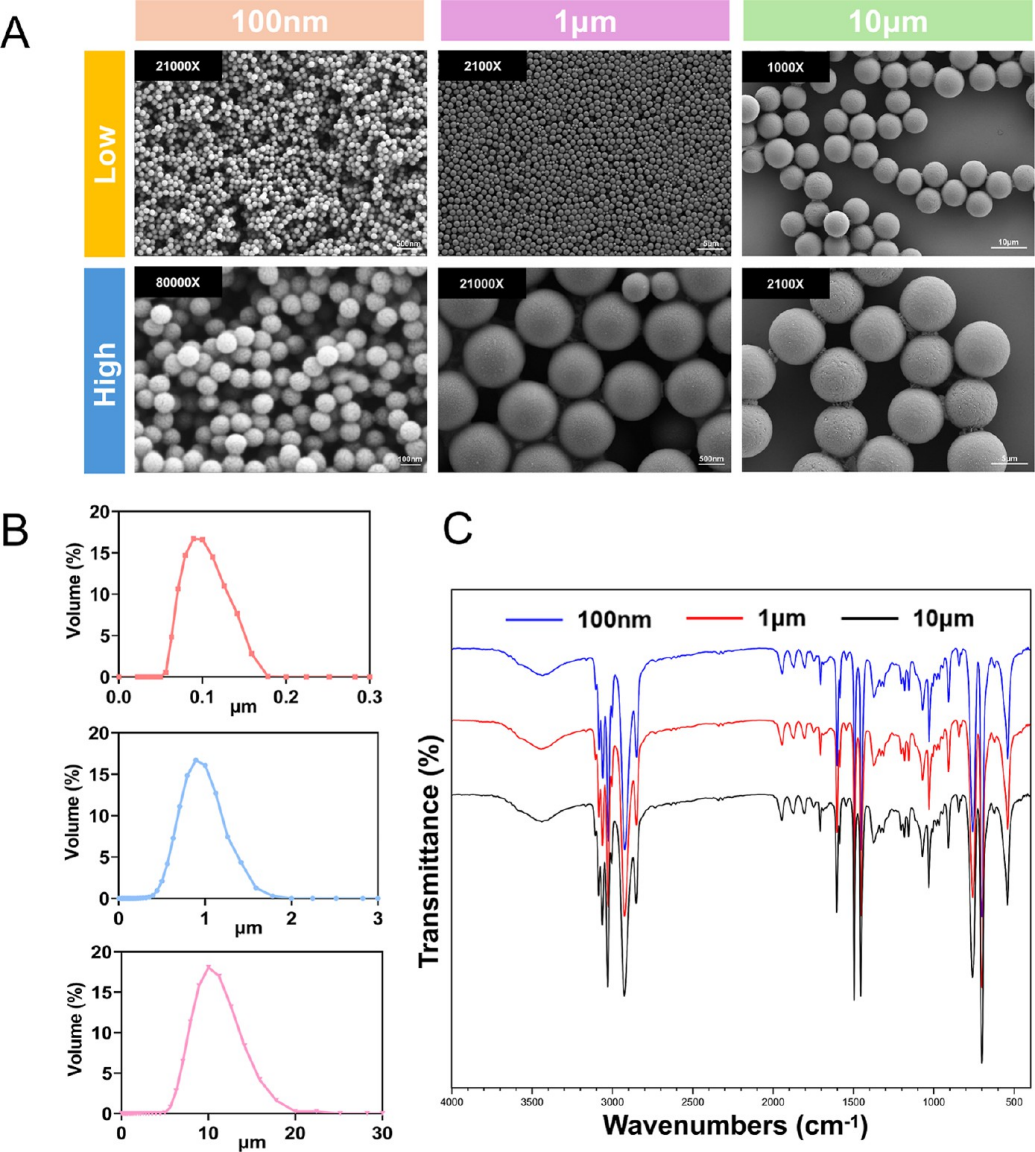
Characterization of PS MPs. (A) SEM images of PS MPs with three different diameters (100 nm, 1 μm, and 10 μm) shown under both low and high magnification. (B) Laser particle size analyzer detection of the average size and distribution of PS MPs with three different diameters. (C) FTIR spectral analysis of PS MPs.

### 3.2 Cell Uptake and In Vivo Distribution and Accumulation of PS MPs

The characterization of PS MPs demonstrated their excellent experimental properties. To evaluate the phagocytic capacity of colonic epithelial cells for PS MPs of varying sizes, NCM460 cells were cultured in vitro and coincubated with PS MPs of three different sizes for 24 h. The results indicated that 100 nm PS MPs were most readily engulfed by colonic epithelial cells, followed by 1 μm PS MPs, while 10 μm PS MPs could not be engulfed (Figures 2A, S1).

**Figure 2:**
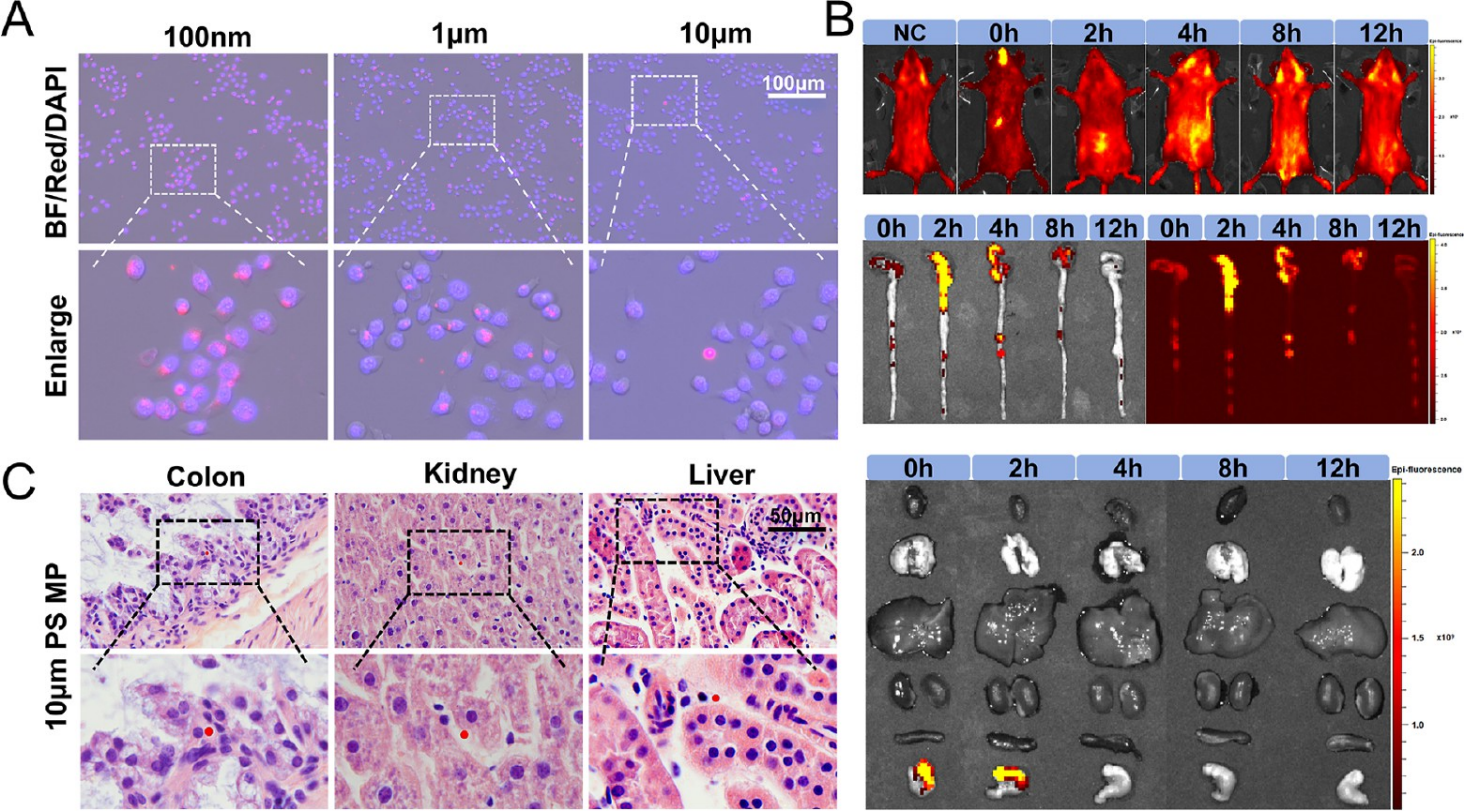
Cell uptake and in vivo distribution and accumulation of PS MPs. (A) The cellular uptake of red fluorescent PS MPs with three different particle sizes coincubated with NCM460 cells for 24 h was examined. BF: bright field channel, Red: red fluorescence channel, DAPI: DAPI channel. BF/Red/DAPI: merged image. Enlarge: enlarged image. Scale bar: 100 μm. (B) Mice were subjected to single high-dose oral exposure to 10 μm red fluorescent PS MPs, and live imaging of their bodies and various organs was conducted at 0 h, 2 h, 4 h, 8 h, and 12 h postexposure. (C) Long-term exposure of mice to 10 μm red fluorescent PS MPs was investigated. H&E-stained tissue sections of the intestine, liver, and kidney were observed under a fluorescence microscope using both bright-field and fluorescence channels, and the images were subsequently merged. Scale bar: 50 μm.

To further investigate the uptake and distribution of 10 μm PS MPs following a single high-dose oral exposure (0–12 h) in vivo, small animal live imaging technology was used to simulate the digestion process of PS MPs in the gastrointestinal tract after ingestion by mice and explore their distribution across various tissues and organs at different time points (Figure 2B). The live imaging results showed that at 0 h, strong fluorescence signals from red fluorescent PS MPs were detected in the stomach immediately after gavage, with no notable signals in the intestines or other organs. At 2 h, PS MPs had reached the midlower abdomen, with significant fluorescence signals detected in the cecum and parts of the colon, while undigested PS MPs were still present in the stomach. By 4 h, the fluorescence signal in the stomach disappeared, suggesting the possible complete digestion of the PS MPs, while the MPs were primarily distributed in the left abdomen, with bright fluorescence signals observed in the left colon and rectum. At 8 h, as PS MPs were gradually excreted, the fluorescence signal in the abdomen diminished, accompanied by a decrease in fluorescence signals in the cecum and colon. A pronounced increase in fluorescence signal was observed around the anus. By 12 h, the overall fluorescence signals in the abdominal area significantly decreased, indicating that most PS MPs had been metabolized and excreted from the body. Furthermore, during this single high-dose oral exposure, no fluorescence signals were detected in organs beyond the gastrointestinal tract. In vivo imaging confirmed that single high-dose oral exposure to PS MPs primarily affected the gastrointestinal tract, with no evidence suggesting that distant organs were invaded by PS MPs.

However, long-term exposure to PS MPs in vivo led to accumulation in distant organs. After 6 weeks of gavage with red fluorescent PS MPs, histological tissue sections revealed red fluorescent particle accumulation in the colon, liver, and kidneys (Figure 2C). These results suggest that prolonged exposure to PS MPs may not only result in accumulation within the intestines but also lead to the accumulation of MPs in multiple organs.

### 3.3 Long-Term Exposure to PS MPs Results in Colonic Inflammation and Oxidative Stress

Following prolonged exposure to PS MPs, changes in mouse body weight were first observed (Figures 3B,C, S2A). The body weight growth curve indicated no significant difference between the MP and NC groups before the initiation of gavage (*P* > 0.05). However, during the gavage period, the body weight gain rate in the MP group was notably slower than that in the NC group. By the end of the gavage period, the body weight of mice in the MP group was significantly lower than that of the NC group (*P* < 0.05). After euthanizing the mice and collecting specimens, comparisons of colonic length and weight revealed that both were significantly reduced in the MP group compared to the NC group (*P* < 0.05) (Figure 3A,B). Furthermore, H&E staining of the MP group showed significant infiltration of inflammatory cells (Figure 3D). ELISA results showed that pro-inflammatory cytokines TNF-α, IL-1β, and IL-6 were significantly higher in the MP group compared to the NC group, while the anti-inflammatory cytokine IL-10 was significantly reduced (*P* < 0.05) (Figure 3E). These results suggest that prolonged exposure to PS MPs slowed weight gain in mice and induced colonic inflammation.

**Figure 3:**
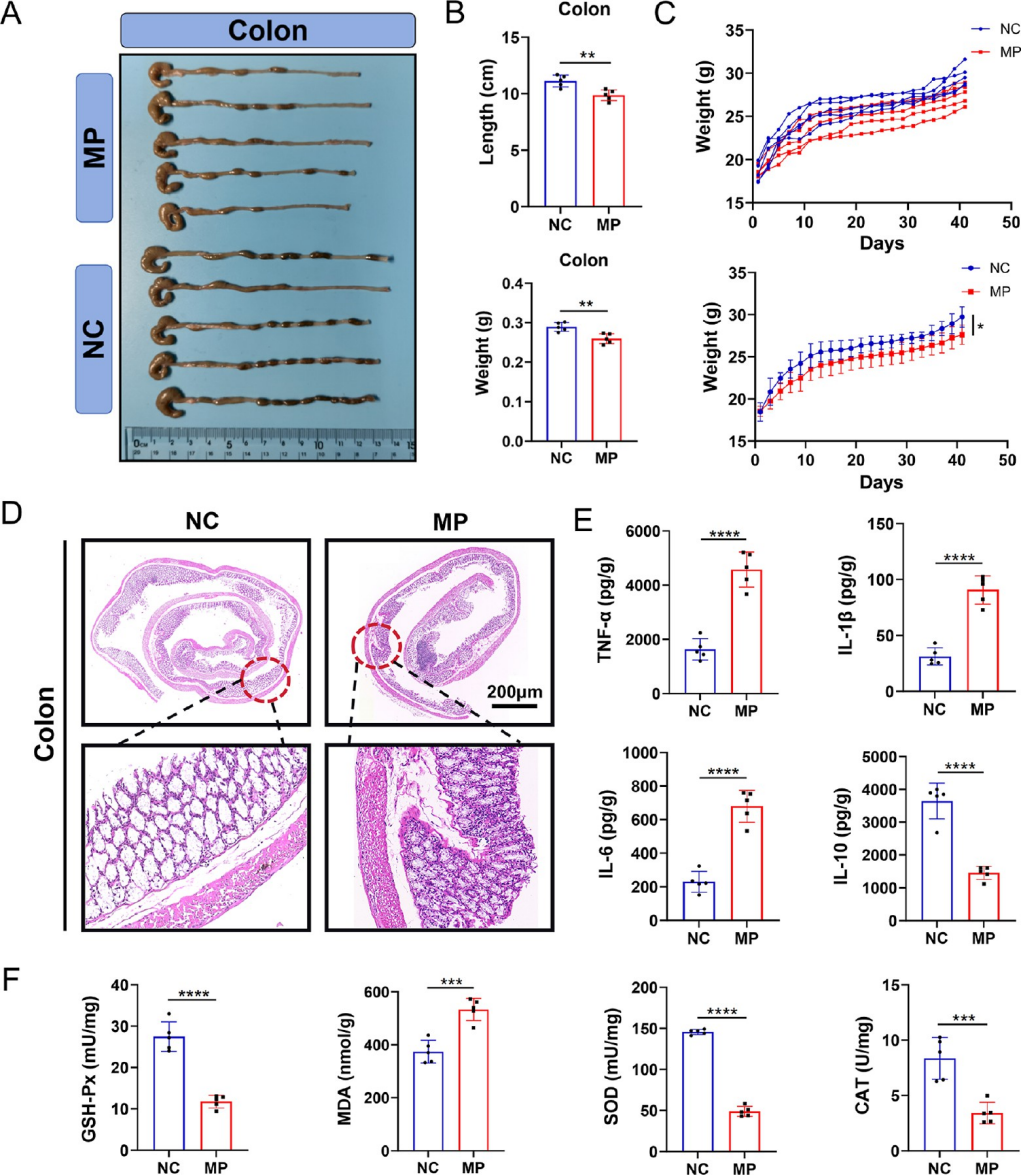
Long-term exposure to PS MPs results in colonic inflammation and oxidative stress. (A) Comparison of colonic specimen lengths between the NC group and the MP group after prolonged exposure to PS MPs. (B) Comparison of colonic length and weight between the NC group and the MP group. (C) Changes in body weight growth curves of individual mice and groups during gavage with PS MPs in the NC group and the MP group. (D) H&E staining of colonic tissues in the NC group and the MP group. (E) Expression levels of TNF-α, IL-1β, IL-6, and IL-10 in the NC group and the MP group detected by ELISA. (F) Expression levels of oxidative stress markers GSH-Px, MDA, SOD, and CAT in colonic tissues. *p < 0.05, **p < 0.01, ***p < 0.001, ****p < 0.0001, *n* = 5.

Analysis of oxidative stress markers in colonic tissues (Figures 3F, S2B) showed that prolonged exposure to PS MPs significantly increased the levels of GSSG and MDA, while GSH levels were significantly decreased in the MP group (*P* < 0.05). Additionally, the expression levels of antioxidant enzymes GSH-Px, SOD, and CAT were significantly lower in the MP group compared to the NC group (*P* < 0.05). These results indicate that prolonged exposure to PS MPs caused an imbalance in the oxidative–reductive system of the mouse colon, leading to oxidative stress-induced damage to colonic tissues.

### 3.4 PS MPs Disrupt the Th17/Treg Balance and Damage the Intestinal Mucosal Barrier in Mice

Th17/Treg cells contribute to the development of intestinal inflammation. To investigate whether PS MPs-induced colonic inflammation is linked to changes in the Th17/Treg ratio in mice, flow cytometry was employed to assess the ratio of Th17 and Treg cells in the spleen (Figures 4A, S3). Quantitative analysis of CD4+ IL17A+ (Th17) and CD4+ CD25+ Foxp3+ (Treg) cells within spleen CD4+ T cells revealed that, compared to the control group, PS MPs significantly increased Th17 cell levels and decreased Treg cell levels, resulting in a notable elevation of the Th17/Treg ratio (*P* < 0.05). These results suggest that PS MPs exposure selectively increases Th17 levels while reducing Treg levels, leading to a disruption of intestinal immune homeostasis.

**Figure 4:**
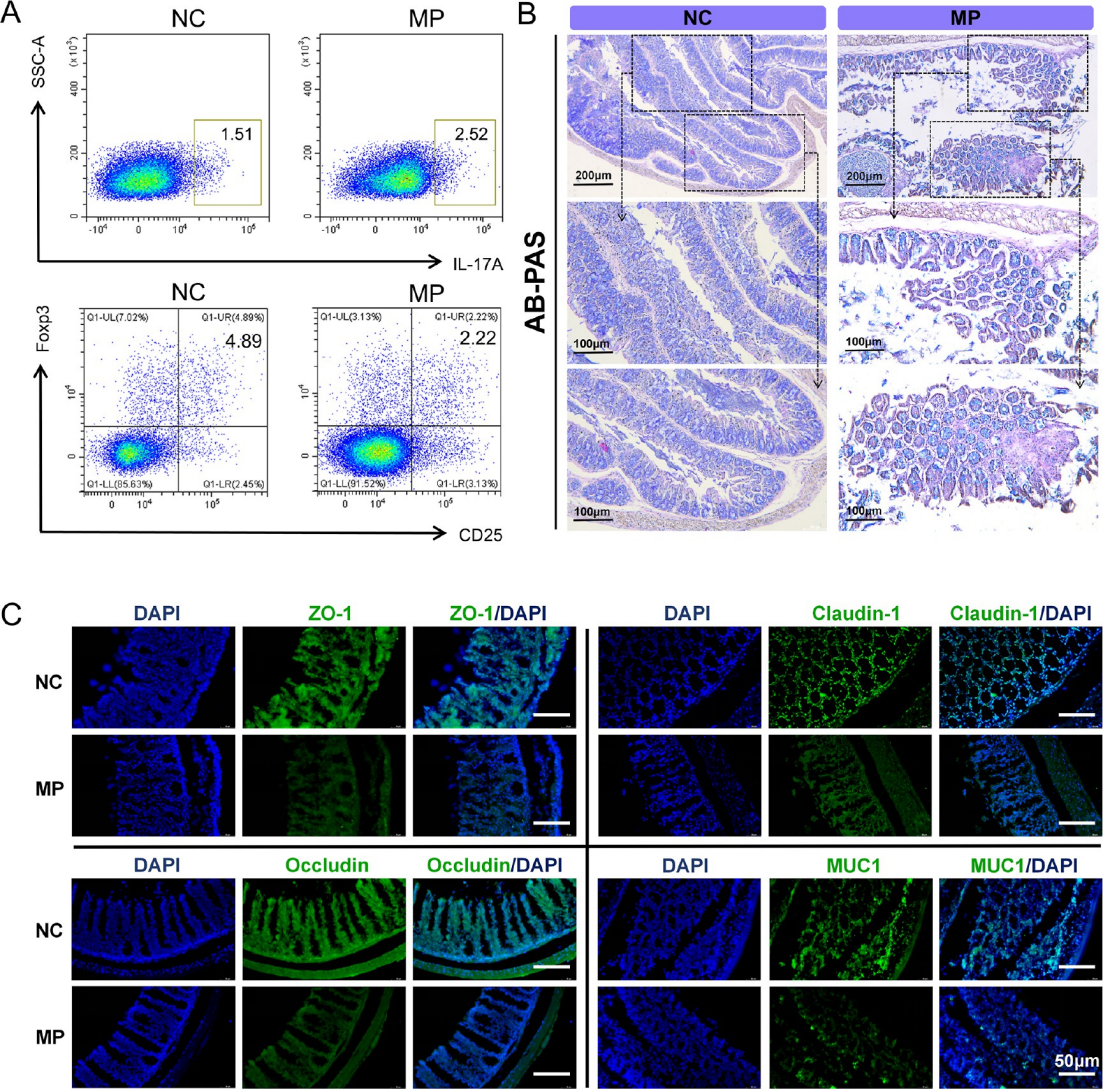
PS MPs disrupt the Th17/Treg balance and damage the intestinal mucosal barrier in mice. (A) Flow cytometry analysis of the percentages of splenic CD4+ IL17A+ (Th17) and CD4+ CD25+ Foxp3+ (Treg) cells. (B) AB-PAS staining of colonic tissues to detect colonic mucus, with mucins displayed as blue or bluish-purple. (C) Immunofluorescence assessment of ZO-1, Occludin, Claudin 1, and MUC1 protein expression levels in colonic tissues. *n* = 3.

The intestinal mucosal epithelial barrier is a primary protective mechanism that shields the gut from damage by harmful stimuli. MPs may compromise this barrier, potentially triggering inflammation. AB-PAS staining showed that the MP group exhibited a reduction in goblet cells within the colonic mucosa, accompanied by decreased mucin secretion, leading to a reduced mucin coverage area in the colon compared to that in the control group (Figure 4B). Additionally, immunofluorescence (IF) staining of colon tissues was used to assess the expression of intestinal barrier proteins (Figure 4C). The results demonstrated a decrease in fluorescence intensity and lower expression levels of ZO-1, Occludin, Claudin 1, and MUC1 in the MP group.

In conclusion, long-term exposure to PS MPs results in colon oxidative stress, an imbalance in the Th17/Treg immune response, extensive infiltration of inflammatory cells in colon tissues, increased secretion of pro-inflammatory cytokines, and disruption of the intestinal epithelial barrier. These results suggest that PS MPs exposure contributes to dysregulation of intestinal homeostasis and induction of colonic inflammation in mice.

### 3.5 PS MPs Induce Liver Damage and Abnormal BA Secretion

The long-term effects of PS MPs exposure on distant organs, such as the liver, were also investigated. Figure 5A presents specimens from the NC and MP groups, including the heart, liver, spleen, lungs, kidneys, and stomach. No significant differences in the appearance of these organs were observed between the two groups. However, upon weighing the organs, no significant differences were found in the masses of the heart, lungs, and stomach (*P* > 0.05). In contrast, the liver and spleen mass were significantly increased, while the kidney mass was significantly decreased in the MP group compared to the control group (*P* < 0.05) (Figures 5B, S4). In addition to the intestine, the liver is one of the most commonly studied distant organs. H&E staining of liver tissue sections (Figure 5C) revealed inflammatory damage in the liver of the MP group. Furthermore, the activities of hepatic antioxidant enzymes, including GSH-Px, SOD, and CAT, were significantly reduced in the MP group (Figure 5E). A significant increase in TBA was also detected in the liver of the MP group (Figure 5D). These findings, combined with the results from Figure 2C, suggest that long-term PS MPs exposure leads to their accumulation in the liver, causing inflammatory damage, impairing antioxidant function, and disrupting BA metabolism.

**Figure 5:**
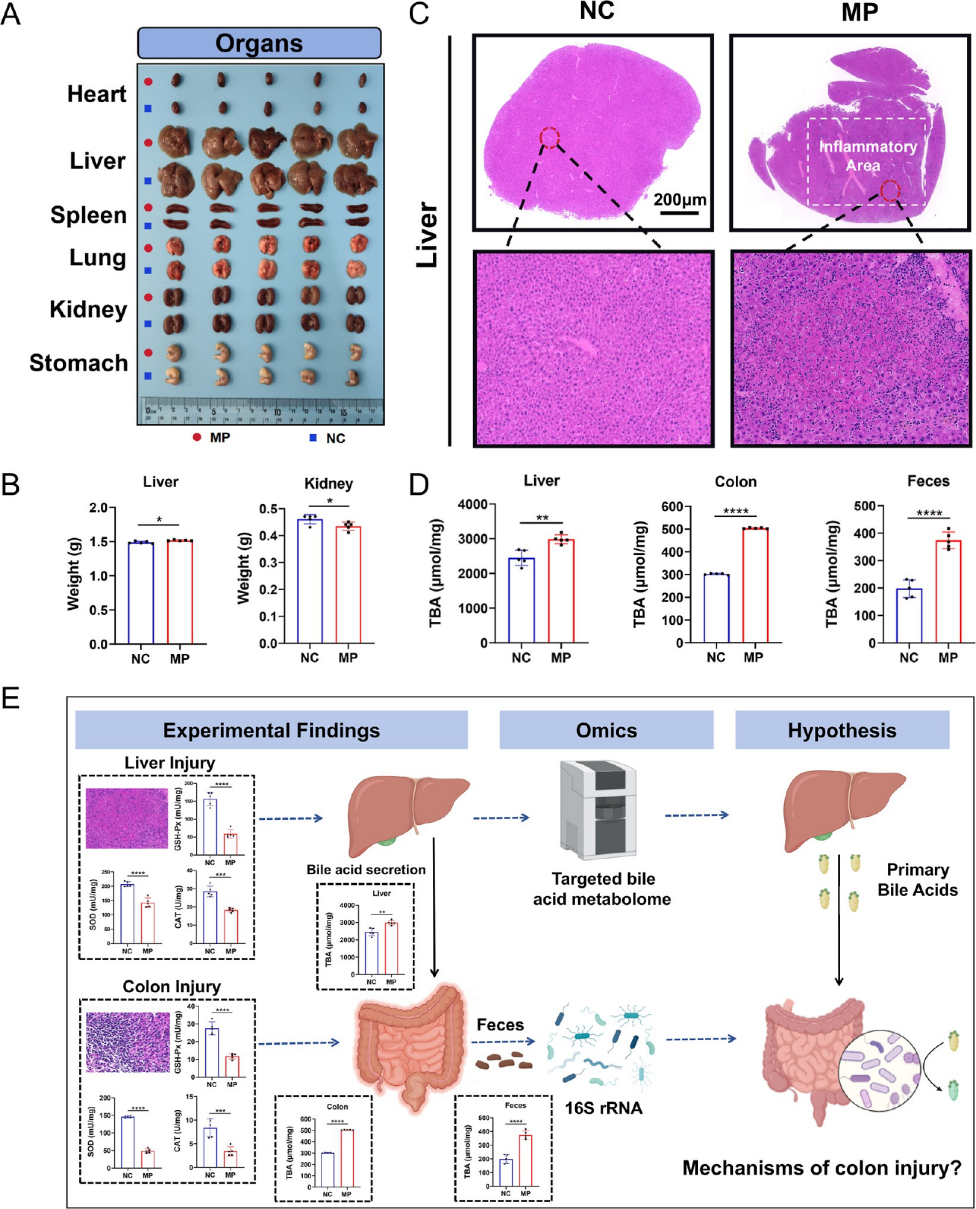
PS MPs induce liver damage and abnormal BA secretion. (A) Comparison of specimens from the heart, liver, spleen, lungs, kidneys, and stomach between the two groups. (B) Statistical analysis of the weights of specimens from the liver and kidneys between the two groups. (C) H&E staining of liver tissues from the NC group and MP group. (D) ELISA detection of TBA levels in the liver, colon, and feces from both groups. (E) Schematic diagram illustrating the proposed mechanism of colonic inflammation and damage induced by long-term exposure to PS MPs. *p < 0.05, **p < 0.01, ****p < 0.0001, *n* = 5.

Additionally, TBA levels in the colon and feces were significantly elevated in the MP group compared to the control group (*P* < 0.05) (Figure 5D). This suggests that the elevated hepatic TBA levels in the MP group result in increased secretion of TBA into the intestine, which, in turn, raises the fecal TBA levels. The mechanism underlying colonic inflammation and damage induced by PS MPs exposure may be related to elevated BA levels. Intestinal microbiota, which can convert primary BAs into secondary BAs, may play a pivotal role in this process. To explore this further, multiomics analysis of fecal BA metabolism and changes in intestinal microbiota were performed using BA metabolism metabolomics and 16S rRNA microbial sequencing technologies (Figure 5E).

### 3.6 Fecal BA Metabolomics Indicates PS MPs-Induced Abnormal Hepatic BA Secretion

BA metabolomic sequencing was conducted on fecal samples from the previously mentioned experiments. Principal component analysis (PCA) of fecal samples from both groups revealed that long-term exposure to PS MPs significantly altered the overall structure of fecal BAs, leading to notable differences in overall metabolism and increased variability (Figure 6A). Subsequently, cluster heatmap analysis was performed on all samples from both groups to observe changes in various BAs, as shown in Figure 6B. Using cluster analysis and the orthogonal partial least squares-discriminant analysis (OPLS-DA) model, variable importance in projection (VIP) scores were obtained. Differential BAs were selected based on *P*-values and fold change (FC) values from univariate analysis. Figure 6C highlights the top 20 differential BAs in terms of FC between the NC and MP groups, with the majority of BAs being elevated in the MP group. To further analyze the patterns of BA changes, the original levels of the selected differential BAs were normalized using unit variance scaling (UV scaling). A total of 17 differential BAs were identified, with 14 upregulated and 3 downregulated. Cluster heatmap analysis of these differential BAs is presented in Figure 6D. Additionally, violin plots were used to visually display the differences in levels and the overall distribution of differential BAs between the two groups (Figures 6E, S5). These results clearly demonstrate the global disruption of BA metabolism induced by prolonged PS MPs exposure. Fecal BAs were predominantly elevated, with 14 out of 17 differential BAs (82.4%) showing significant increases, which aligns with enhanced hepatic synthesis and secretion (refer to liver and colon TBA data, Figure 5D). Among the 14 upregulated BAs, 11 were conjugated types (78.6%), with taurine-conjugated BAs predominating (8 out of 11, 72.7%). Furthermore, this abnormal BA profile may be linked to impaired gut microbiota functionality, suggesting the need for further investigation to elucidate the underlying mechanism.

**Figure 6:**
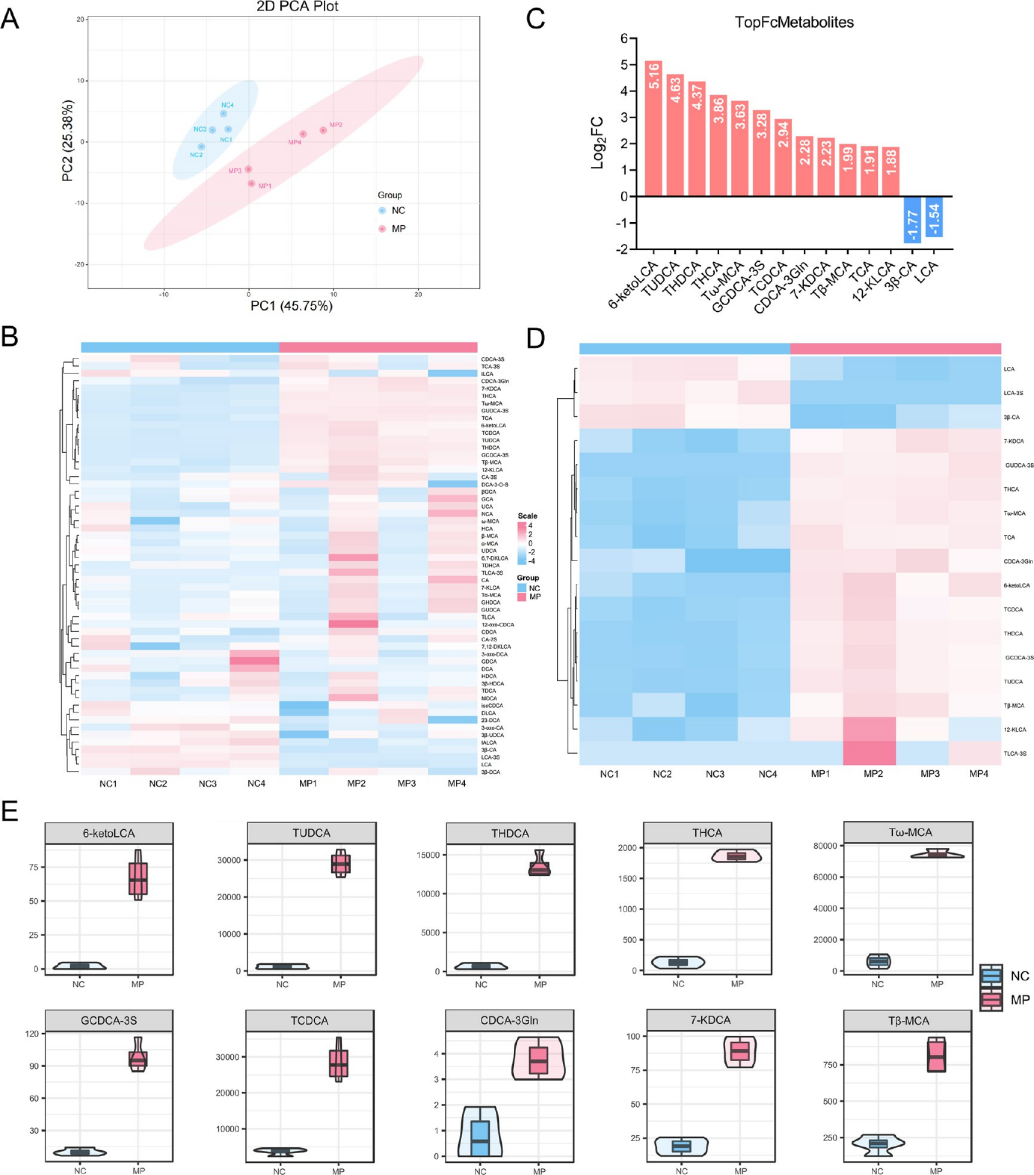
Fecal BA metabolomics indicates PS MPs-induced abnormal hepatic BA secretion. (A) 2D score plot from PCA of bile acids. (B) Cluster heatmap analysis of BA data. (C) Bar plot displaying the top 20 differentially expressed bile acids. Red represents upregulated bile acids, while blue represents downregulated bile acids. (D) Cluster heatmap analysis of differential bile acids. (E) Violin plot of differential bile acids. *n* = 4.

### 3.7 Alterations of the Gut Microbiota in the PS MPs Group

In 16S rRNA sequencing, the OTUs were identified through clustering methods, and a Venn diagram was generated. The analysis revealed 724 OTUs shared between the NC and MP groups, with 372 unique to the NC group and 397 unique to the MP group (Figure 7A). Further investigation of species with high relative abundance at each taxonomic level and their proportional changes was conducted based on OTUs (Figures 7B, S6A). The results demonstrated that PS MPs exposure induced multilevel shifts in the microbiota. At the phylum level, Firmicutes and Actinobacteriota were significantly enriched, while Bacteroidota, Proteobacteria, and Verrucomicrobiota were markedly depleted. These phylum-level alterations were driven by dynamic changes in the key families and genera. Firmicutes enrichment correlated with increased abundance of Lachnospiraceae and its associated genera and . In contrast, Bacteroidota depletion was linked to reduced levels of Bacteroidaceae and its dominant genus Bacteroides. At the species level, analysis revealed a bifurcation within the Lactobacillus genus: and were upregulated, while and the mucin-degrading species were suppressed. These taxon-specific responses suggest the selective regulation of microbial consortia involved in mucin metabolism and immunomodulation by PS MPs. To assess microbial diversity and community structure, a multiple sequence alignment of the top 100 genera was performed, and their phylogenetic relationships were inferred (Figure S6C). Principal component analysis (PCA) and principal coordinate analysis (PCoA) revealed significant alterations in the intestinal microbiota composition induced by PS MPs (Figure 7C). Simper analysis identified key taxa with intergroup differences, including Firmicutes, Bacteroidota, and Proteobacteria at the phylum level, as well as , , and at the genus level (Figure S6B). LEfSe analysis further highlighted 8 bacterial taxa significantly affected by PS MPs (Figure S6D).

**Figure 7:**
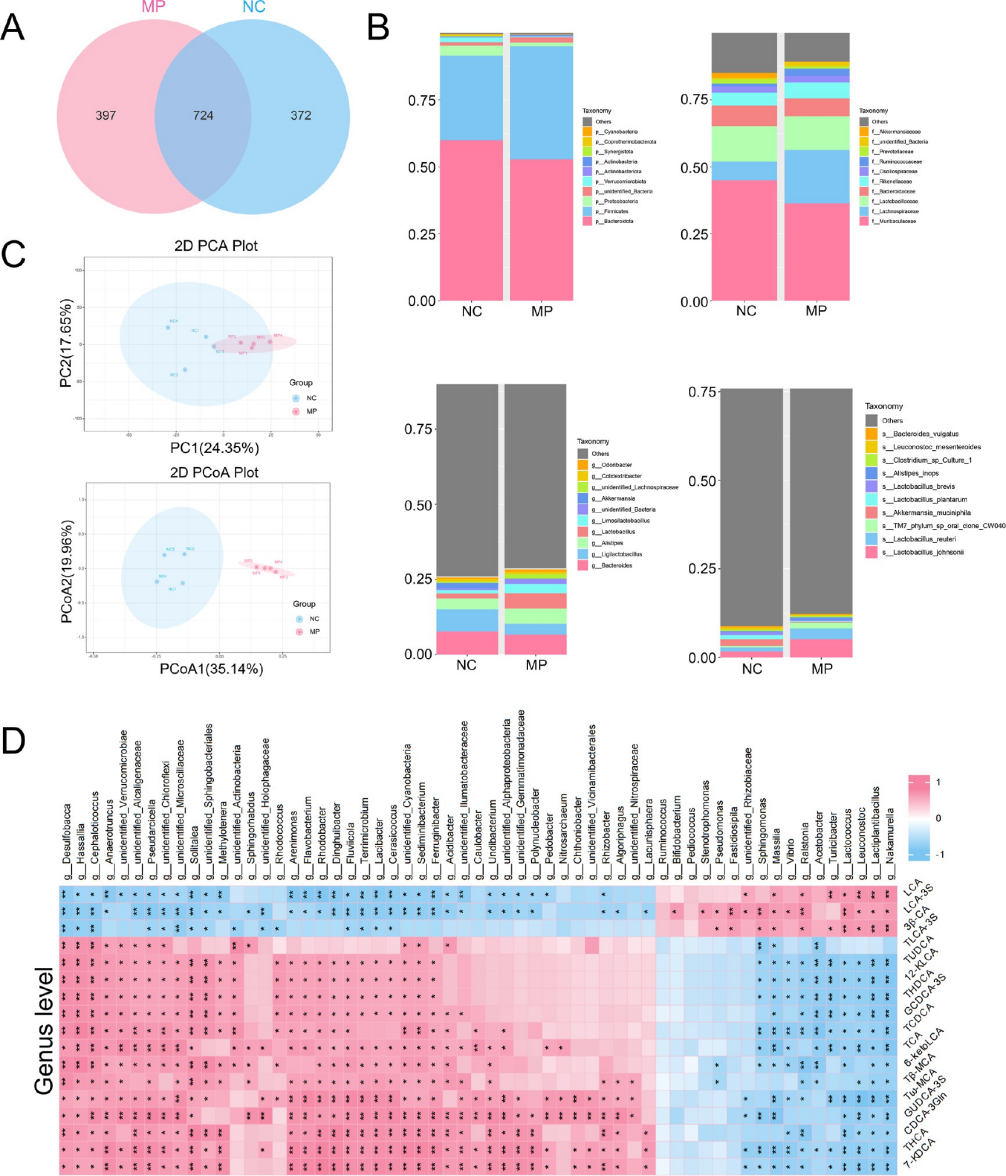
Alterations of the gut microbiota in the PS MPs group. (A) Venn diagram based on OTUs. (B) Composition of intestinal microbiota at the phylum, family, genus, and species levels. (C) PCA score plot and PCoA score plot. (D) Spearman correlation clustering heatmap at the genus level showing the differential microbial communities in mice after PS MPs treatment and their correlation with differential bile acids. Red: positive correlation; blue: negative correlation. *p < 0.05, **p < 0.01, *n* = 4.

To further elucidate the interaction between gut microbiota and BAs under PS MPs exposure, Spearman correlation analysis was performed on differential microbiota and BA metabolites (Figures 7D, S7A,B). The results (*P* < 0.05) revealed significant positive correlations between elevated BAs and Actinobacteria at the phylum level, while negative correlations were observed with Proteobacteria. At the genus level, BAs positively correlated with potentially harmful bacteria such as and but negatively correlated with beneficial genera including , , and . Species-level analysis further showed that increased BA levels were positively associated with oxidative stress- and inflammation-inducing pathogens like , while negatively correlated with probiotics such as , , and , which play roles in maintaining barrier integrity, exerting anti-inflammatory effects, and regulating metabolism. These results suggest that PS MPs exposure may exacerbate colonic inflammation and injury by disrupting the correlation between specific microbiota and BAs, leading to the enrichment of potential pathogens and reduction of beneficial bacteria.

### 3.8 TCDCA Exacerbates PS MPs-Induced Apoptosis in Colon Epithelial Cells

BA metabolism disorders are characterized by a significant elevation in conjugated BA levels, particularly tauro-conjugated species. To further investigate the role of BAs in PS MPs-induced colitis and intestinal injury, TCDCA, a representative tauro-conjugated BA, was selected for this study. CCK-8 assays were conducted to determine optimal treatment concentrations, revealing that PS MPs (0.5–2 mg/mL) and TCDCA (≥1200 μM) induced dose-dependent cytotoxicity in NCM460 cells (Figures 8A, S8A). At 0.5 mg/mL, PS MPs reduced cell viability to 88.43%, while concentrations ≥2 mg/mL resulted in near-total cell death. TCDCA ≥1200 μM also exhibited significant toxicity. To minimize PS MPs-induced mortality while examining TCDCA’s role, 0.5 mg/mL PS MPs were combined with graded concentrations of TCDCA. The combination of 0.5 mg/mL PS MPs with 1000 μM TCDCA resulted in severe cytotoxicity (13.22% viability). The experimental groups included NC (control), MP (0.5 mg/mL PS MPs), TCDCA (1000 μM), and MP + TCDCA (combined treatment).

**Figure 8:**
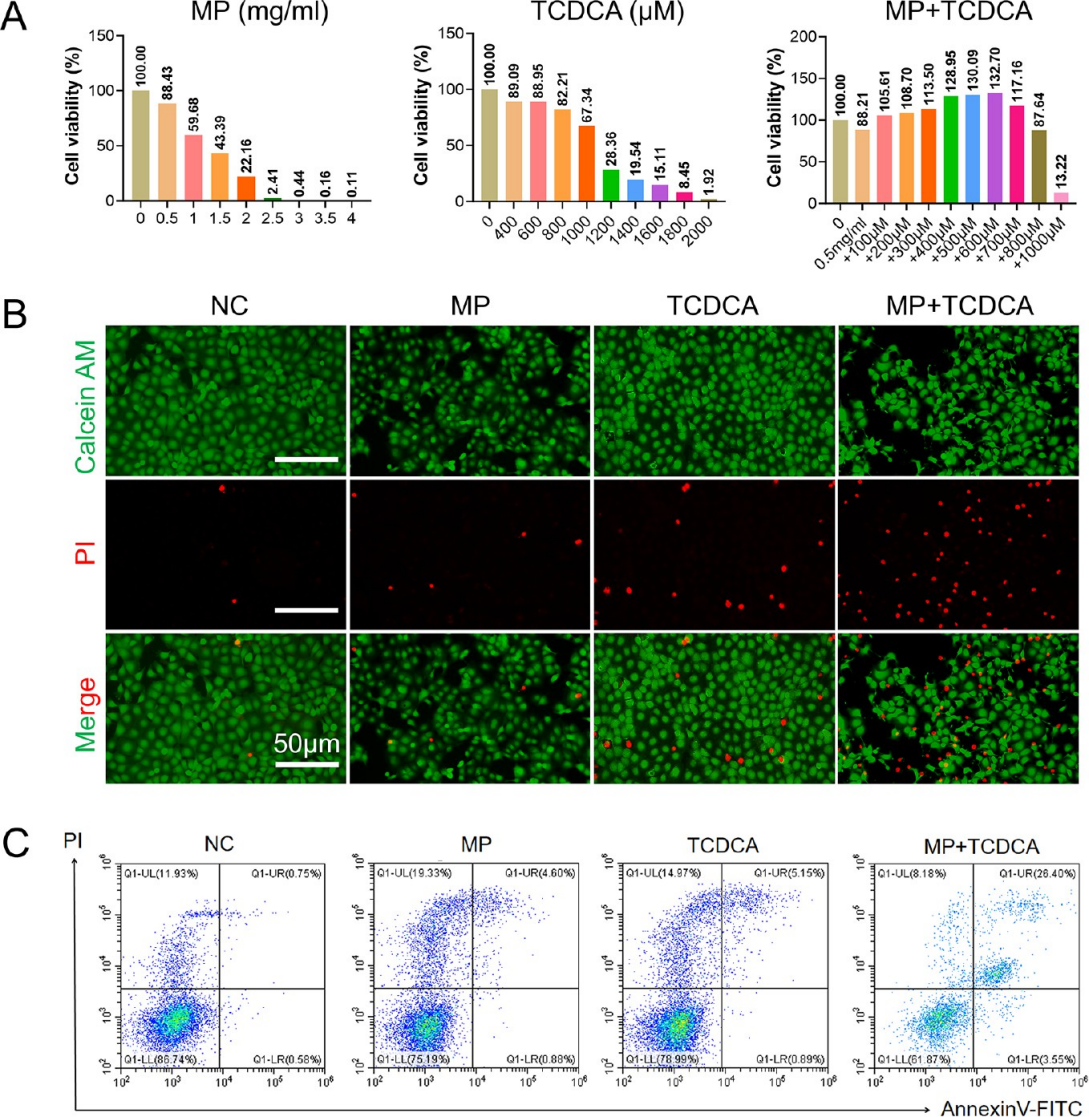
TCDCA exacerbates PS MPs-induced apoptosis in colon epithelial cells. (A) CCK-8 cell proliferation and toxicity assay. (B) Calcein-AM/PI live/dead cell fluorescence staining. Scale bar: 50 μm. (C) Annexin V-FITC flow cytometry apoptosis detection. *n* = 3.

Figure 8B presents Calcein-AM/PI staining results where live (green) and dead (red) cells reflect the degree of damage. The NC group exhibited minimal red fluorescence, indicating normal cell viability. The MP group showed slight red signals, while TCDCA treatment increased red fluorescence moderately. Notably, the MP + TCDCA combination induced a dramatic increase in red signals, demonstrating synergistic cytotoxicity. ROS analysis (Figure 9A) revealed negligible green fluorescence in control cells, whereas intense signals were observed in Rosup-treated cells. MP exposure induced mild ROS generation, which was further enhanced by TCDCA. The combination group exhibited the strongest fluorescence, indicating significant ROS overproduction. JC-1 staining (Figure 9B) was used to assess mitochondrial membrane potential, with red/green fluorescence shifts indicating changes. NC cells showed predominant red signals (intact mitochondria), while CCCP-treated cells displayed a green dominance. Both MP and TCDCA treatments resulted in reduced red fluorescence compared with NC, with TCDCA showing slightly stronger green signals. The combination group showed near-complete conversion to green fluorescence, indicating severe mitochondrial dysfunction.

**Figure 9:**
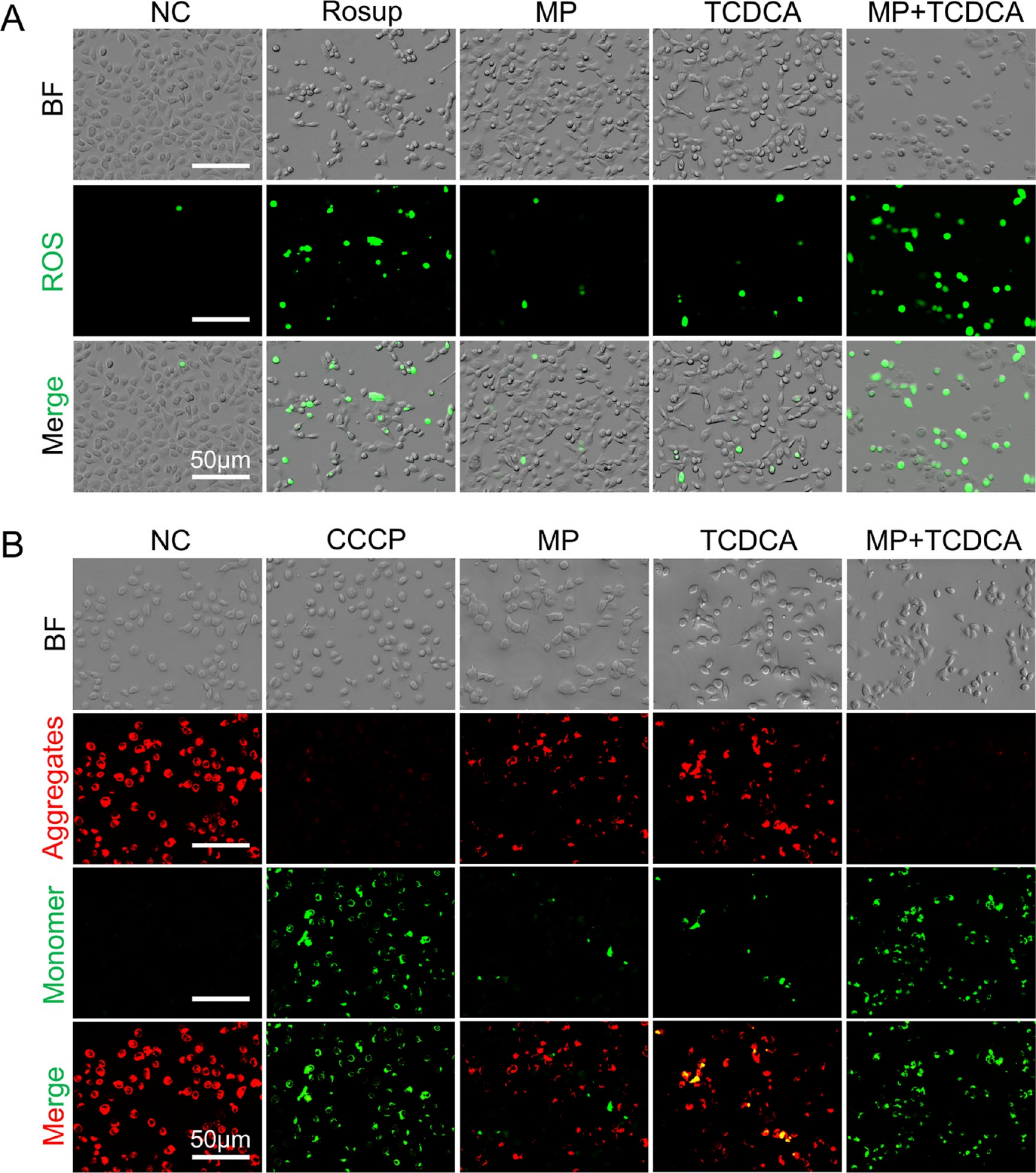
TCDCA exacerbates PS MPs-induced apoptosis in colon epithelial cells. (A) ROS detection. (B) JC-1 mitochondrial membrane potential detection. BF: bright-field view, Merge: multichannel overlay image. Scale bar: 50 μm, *n* = 3.

Subsequent flow cytometry and Western blot analyses provided further insights into apoptosis regulation. Figure 8C demonstrates an increase in apoptosis across groups: NC (1.33%), MP (5.48%), TCDCA (6.04%), and MP + TCDCA (29.95%), indicating synergistic pro-apoptotic effects. Western blot analysis of mitochondrial apoptosis markers (Figure S8B) showed consistent patterns when normalized to GAPDH. Pro-apoptotic proteins Bax, caspase-9, caspase-3, and PARP exhibited low expression in the NC group, increased in the MP and TCDCA groups, and reached the highest levels in the MP + TCDCA group, showing significant upregulation. Conversely, the antiapoptotic protein Bcl-2 exhibited the highest expression in the NC group, decreased in the MP and TCDCA groups, and was lowest in the MP + TCDCA group, demonstrating significant downregulation. These molecular changes align with the flow cytometry results, confirming that TCDCA significantly enhances PS MPs-induced apoptosis through activation of the mitochondrial pathway.

### 3.9 TCDCA Exacerbates PS MPs-Induced Colitis and Intestinal Injury in Mice

In vitro findings indicated that TCDCA exacerbates PS MPs-induced colonic epithelial damage, leading to subsequent in vivo validation. Broad-spectrum antibiotics (ABX) were used to deplete the gut microbiota, allowing for further investigation into how the microbiota–metabolite interaction influences colitis progression. Six-week murine experiments showed significant reductions in colonic length and weight in the MP group compared to NC controls (*P* < 0.05). Depletion of gut microbiota through ABX (MP + ABX group) mitigated these morphological changes, with significantly greater colonic length and weight observed compared to the MP group (*P* < 0.05). Conversely, TCDCA administration (MP + TCDCA group) exacerbated PS MPs-induced damage, with further reductions in colonic parameters compared to the MP group (*P* < 0.05, Figures 10A,B, S9A).

**Figure 10:**
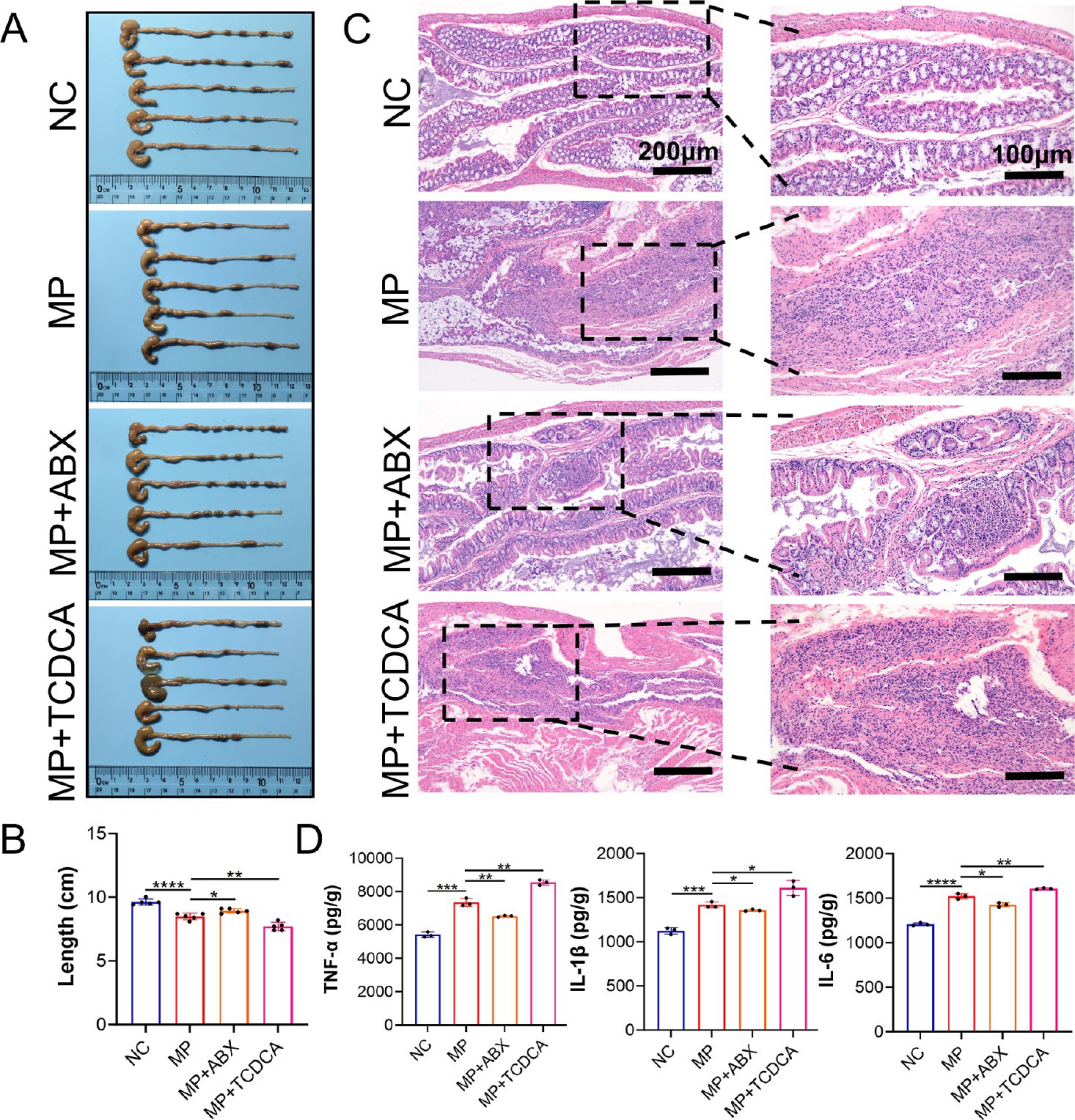
TCDCA exacerbates PS MPs-induced colitis and intestinal injury in mice. (A) Comparison of colon length in different groups of mice. *n* = 5. (B) Statistical analysis and comparison of colon length in different groups of mice. *n* = 5. (C) H&E pathological staining of mouse colon tissue. *n* = 3. (D) ELISA detection of expression levels of inflammatory factors TNF-α, IL-1β, IL-6 in mouse colon. *n* = 3.*p < 0.05, **p < 0.01, ***p < 0.001, ****p < 0.0001.

Histopathological analysis (Figure 10C) revealed progressive mucosal alterations in the experimental groups. The NC group maintained intact mucosal architecture with well-organized intestinal villi and distinct crypt structures. MP exposure led to significant pathological changes, including villous atrophy, crypt disorganization, and substantial submucosal inflammatory infiltration, accompanied by vascular congestion and edema. Microbiota-depleted (MP + ABX) animals exhibited partial preservation of mucosal structures, with relatively ordered villi/crypt arrangements and attenuated inflammatory manifestations compared to the MP group. The MP + TCDCA group, however, exhibited severe mucosal destruction characterized by epithelial necrosis, total villous/crypt loss, and exacerbated submucosal vascular abnormalities with dense inflammatory infiltrates. ELISA analysis of inflammatory mediators (Figures 10D, S9B) revealed distinct cytokine profiles. Pro-inflammatory cytokines (TNF-α, IL-1β, IL-6) were elevated in the MP, MP + ABX, and MP + TCDCA groups compared to NC (*P* < 0.05), with the MP + ABX group showing significant reduction and the MP + TCDCA group exhibiting maximal elevation compared to MP controls. Conversely, the anti-inflammatory cytokine IL-10 displayed an inverse pattern, with suppressed levels across all treatment groups (*P* < 0.05), partially restored in MP + ABX and further diminished in MP + TCDCA.

The integrated in vitro and in vivo findings demonstrate that TCDCA exacerbates PS MPs-induced colitis and intestinal injury in mice, with its interaction with the gut microbiota likely playing a critical role in the aggravated toxic effects of PS MPs.

## 4 Discussion

Particle size is a critical determinant of biodistribution and toxicity.^12^ While the effects of MPs of varying sizes on the intestines have been extensively studied, the mechanisms underlying intestinal toxicity induced by larger sized NPMs remain relatively underexplored, warranting further investigation. The diameter of human intestinal epithelial cells typically ranges from 10 to 30 μm, and their phagocytic activity is generally limited to the uptake of MPs smaller than 5 μm.^29^ Previous research has established 10 μm as the upper limit for cellular uptake of MPs.^10^ A study conducted in Germany also demonstrated that MPs of 10 μm or larger exceed the size threshold for cellular uptake.^30^ In vitro simulated digestion experiments by Ma et al. further confirmed that both NCM460 and Caco-2 intestinal epithelial cells demonstrated minimal absorption of 10 μm particles, whether pristine or digested PS MPs, with these particles being almost entirely nonphagocytosable due to the “oversized effect”.^31^ However, excessively large MPs (>10 μm) not only compromise experimental reliability due to poor monodispersity but also increase the risk of nonspecific physical damage. Therefore, 10 μm PS MPs were selected as the focal subject for this study to better investigate their biological effects. Through a 24 h fluorescent MPs cellular uptake experiment, this study has confirmed that 10 μm PS MPs are nonphagocytosable by intestinal epithelial cells and explored their potential mechanisms in inducing intestinal inflammatory injury in mice.

SEM, laser particle size analysis, and FTIR results confirmed that the PS MPs microspheres used in this study exhibited excellent stability, ensuring the reliability of subsequent research on their intestinal toxicity. The biodistribution of 10 μm fluorescent PS MPs in the digestive system (stomach, small intestine, large intestine), thoracic cavity (heart, lungs), and abdominal organs (liver, kidneys, spleen) of mice was tracked using the IVIS in vivo imaging system and H&E staining under both single and long-term exposure conditions. Under single exposure, 10 μm PS MPs were detected exclusively in the digestive system, with initial detection in the stomach, followed by predominant localization in the small and large intestines over time. This indicates a significant exposure risk to the intestines. It is well-established that mammals possess very few enzymes capable of digesting plastics, making MPs difficult to break down after ingestion.^32^ Consequently, the experimental results also show that, under single exposure, 10 μm PS MPs are less likely to breach the intestinal barrier and invade other tissues extensively, thereby affecting other organs. Despite a high oral gavage dose, the majority of MPs are gradually excreted through feces over time.^33^ Although some fluorescent particles may accumulate in the thoracic and abdominal organs, their concentrations under a single exposure are insufficient for detection. However, long-term exposure to 10 μm PS MPs has been shown to result in the presence of MPs in the intestines, liver, and kidneys of mice, as reported by Deng et al.^2^ Additionally, particles <20 μm can effectively translocate to various organs.^34^ Based on prior studies and preliminary data from this study, the following conclusions can be drawn: following oral exposure to 10 μm PS MPs, the intestines are primarily affected, with the majority of MPs excreted via feces. A small portion may cross the intestinal barrier, enter the bloodstream, distribute throughout the body, and impact other tissues and organs. The remaining MPs may accumulate and adhere to the intestinal mucosa over time, leading to more severe structural and functional intestinal disorders, including damage to the intestinal epithelial barrier, inflammation, immune responses, and gut microbiota dysbiosis.^7–9,23,35^

After 6 weeks of long-term oral exposure to PS MPs, a reduction in body weight and slower growth rate were observed in mice compared to the control group, aligning with previous studies that report inhibited weight gain due to MP exposure.^36^ The colon length was significantly shortened, and H&E pathological staining revealed inflammatory cell infiltration in the intestines. Quantitative ELISA analysis indicated that PS MPs exposure led to a significant increase in pro-inflammatory factors (TNF-α, IL-1β, and IL-6) and a decrease in anti-inflammatory factors (IL-10) in the mouse colon, resulting in an imbalance between pro-inflammatory and anti-inflammatory responses. Pro-inflammatory cytokines such as TNF-α enhance immune responses and inflammation, while the anti-inflammatory cytokine IL-10 counteracts these effects by maintaining immune homeostasis.^37,38^ Despite IL-10s anti-inflammatory effects, under long-term MPs exposure, the levels of pro-inflammatory factors in the intestines significantly exceeded those of anti-inflammatory factors, resulting in an imbalance in the intestinal immune system and triggering inflammatory responses. These findings demonstrate that long-term PS MPs exposure inhibits the growth and development of mice, disrupts intestinal homeostasis, and significantly induces colonic inflammation, likely through multiple mechanisms. However, the specific mechanisms remain unclear, and further investigation is necessary to elucidate the potential pathways by which PS MPs induce colonic inflammatory injury.

Studies have shown that PS MPs can induce the generation of ROS, leading to intestinal oxidative stress and epithelial cytotoxicity, whereas the clearance of ROS can significantly alleviate mucosal damage.^39,40^ These findings indicate that colonic inflammation and mucosal epithelial injury are closely associated with oxidative stress. To assess this, relevant indicators of the colonic redox system were evaluated. The study revealed that after long-term oral exposure to PS MPs, levels of the antioxidant GSH and antioxidant enzymes such as GSH-Px, SOD, and CAT in the mouse colon were significantly reduced, while oxidative products, including GSSG and MDA, were markedly increased. GSH plays a pivotal role in scavenging ROS, while GSH-Px protects cells through both direct and indirect antioxidant mechanisms.^41^ MDA, which is a byproduct of lipid peroxidation, serves as an indicator of oxidative damage. These results demonstrate that long-term exposure to PS MPs disrupts the dynamic equilibrium of the redox system in the mouse colon, triggering oxidative stress that subsequently leads to colonic mucosal epithelial injury and inflammation.

Inflammation is a complex process intricately tied to the body’s immune response, with immune imbalance potentially exacerbating intestinal inflammation. Effector T cells, key coordinators of the immune response, are divided into subsets such as Th1, Th2, Th17, and Treg cells. Among these, Th1 and Th17 cells are pro-inflammatory, while Treg cells are anti-inflammatory.^42^ Under healthy conditions, maintaining dynamic balance between T cell subsets, particularly between Th17 and Treg cells, is essential for immune homeostasis. An imbalance between Th17 and Treg cells is often closely linked to the development of autoimmune and inflammatory diseases.^43^ To explore whether PS MPs exposure influences the Th17/Treg imbalance, the proportions of Th17 and Treg cells within the CD4+ cell population in mouse splenocytes were measured along with the Th17/Treg ratio. The results indicated that long-term PS MPs exposure led to a significant increase in the proportion of Th17 cells, a notable decrease in the proportion of Treg cells, and a marked increase in the Th17/Treg ratio. These findings suggest that after prolonged oral exposure to PS MPs, excessive proliferation of Th17 cells or impaired Treg cell function results in an elevated Th17/Treg ratio, intensifying pro-inflammatory responses, aggravating inflammation, and further exacerbating intestinal injury.

The intestinal barrier comprises three critical components: the mucus layer, tight junctions (TJs), and epithelial cells. This barrier plays a pivotal role in evaluating the intestinal toxicity of MPs and their potential effects on distant tissues and organs. Long-term oral ingestion of MPs can lead to their accumulation in the body via the intestinal barrier, triggering adverse effects such as gut microbiota dysbiosis and metabolic disorders, which may result in multisystem and organ damage.^44^ The intestinal mucus layer is essential for protecting the intestinal mucosa and preventing bacterial invasion, with MUC1, a mucin present on the surface of intestinal epithelial cells, playing a key role in forming this protective barrier.^45^ Our findings indicate that long-term exposure to PS MPs significantly reduces both mucus secretion and MUC1 expression in the mouse colon. This effect is likely related to the significant increase in pro-inflammatory cytokines and the previously discussed imbalance in the redox system. Specifically, TNF-α induces intestinal epithelial cell death, leading to goblet cell loss and impairing mucus production.^46^ Additionally, oxidative stress can degrade mucins, reducing the thickness of the mucus barrier.^47^ TJ proteins, such as ZO-1, claudin, and occludin, are essential in forming intestinal epithelial TJs. These junctions not only maintain the physical and functional integrity of the intestinal barrier but also prevent pathogens and harmful substances from penetrating the intestinal tissue.^48^ ZO-1, a scaffold protein located on the inner side of the cell membrane, connects transmembrane proteins like claudin and occludin to the cytoskeleton, forming a complete TJ complex.^49^ Claudin proteins, key regulators of paracellular permeability, can disrupt intestinal barrier function when altered, promoting inflammatory responses and contributing to the development of necrotizing enterocolitis.^50^ Occludin, a critical transmembrane protein in TJs, plays a significant role in maintaining the barrier function. However, inflammation or oxidative stress can increase its tyrosine phosphorylation, alter its interaction with ZO-1, and disrupt TJ integrity.^51^ The barrier formed by these TJ proteins not only regulates intestinal permeability but also plays a pivotal role in maintaining mucosal immune homeostasis. Therefore, the normal expression and distribution of TJ proteins are vital for maintaining the intestinal barrier’s proper function.^52^ In this study, the expression levels of ZO-1, claudin-1, and occludin proteins were significantly reduced in the PS MPs-treated group compared with the NC group. In summary, long-term oral exposure to PS MPs significantly impairs the intestinal barrier function, resulting in damage to the mucus layer and loss of TJ integrity. This leads to a weakened intestinal epithelial barrier function and increased permeability. Disruption of barrier function may facilitate the penetration and accumulation of PS MPs from the intestinal lumen into the intestinal tissue, inducing intestinal epithelial cell death and further exacerbating intestinal inflammation. Moreover, increased intestinal permeability may allow more PS MPs and other harmful substances to enter the systemic circulation, posing potential toxic risks to distal organs and even the entire system.^53^

BAs are key bioactive molecules synthesized by the liver and are primarily responsible for promoting the digestion and absorption of fats. While most BAs are reabsorbed in the small intestine, a portion enters the colon, where they are metabolized by the gut microbiota. Based on previous research and the experimental findings mentioned above, it has been established that orally ingested PS MPs can cross the intestinal barrier, enter the liver through the bloodstream, and accumulate there. Moreover, intestinal inflammatory responses and barrier damage may further enhance intestinal absorption and hepatic accumulation of PS MPs.^2,23^ Long-term exposure to MPs not only leads to hepatic inflammatory injury but also disrupts lipid metabolism and causes BA dysregulation. Studies have shown that PS MPs can significantly increase hepatic TBA levels, possibly by inducing oxidative stress and inflammatory responses in the liver, thereby promoting the expression of BA synthesis enzymes such as CYP7A1, and by affecting hepatocellular energy metabolism to regulate the function of BA transporters such as BSEP.^23,24,35^ In the present study, PS MPs presence and signs of liver inflammation were observed through liver H&E staining. Concurrently, PS MPs exposure significantly reduced antioxidant enzyme levels, such as GSH-Px, SOD, and CAT, thereby impairing liver function. Furthermore, TBA levels in both the liver and feces of the PS MPs-exposed group were significantly elevated, indicating abnormal secretion of TBA from the liver into the colon. This raises the question: is excessive BA associated with the onset and progression of colitis? Research suggests that a large influx of BAs into the colon may lead to various diseases, including diarrhea, metabolic disorders, and necrotizing enterocolitis.^54^ The cytotoxic mechanisms of BA-induced mucosal damage include detergent effects that disrupt cell membranes and nondetergent effects such as apoptosis.^55,56^ Zhou et al., using a dextran sulfate sodium-induced (DSS)-induced chronic colitis mouse model, found that the PPARα-UGT axis was excessively activated during colitis progression, leading to BA metabolic imbalance.^57^ This resulted in enhanced BA synthesis in the liver. However, due to inflammatory damage compromising the integrity of the colonic mucosal epithelial barrier, toxic BAs abnormally accumulated in the inflamed colon, exacerbating colonic inflammatory injury. Based on the aforementioned studies and our experimental results, BA metabolomic sequencing was performed on mouse feces. The results revealed that PS MPs entering the liver caused BA dysregulation, significantly increasing TBA synthesis and its delivery to the colon, leading to a marked rise in the fecal TBA levels. Analysis of the sequencing data indicated that most differential BAs were upregulated with taurine-conjugated BAs being the predominant type among the upregulated BAs. TCDCA, a high-abundance endogenous BA shared by both humans and mice, was found to exhibit significant biological activity and metabolic relevance, making it a representative and meaningful target for further research. Therefore, TCDCA was selected as the focus for subsequent studies to explore the potential role and mechanisms of elevated BAs in PS MPs-induced colitis.

Gut microbiota dysbiosis (composition and diversity) is closely linked to increased intestinal epithelial barrier permeability and active inflammatory responses in the gut.^58^ MPs exposure induces gut microbiota dysbiosis, characterized by a reduction in beneficial bacteria and an increase in harmful bacteria.^17^ As a key component in maintaining the intestinal epithelial barrier function, gut microbiota dysbiosis not only disrupts intestinal digestion and metabolism but also influences intestinal permeability and immune responses, thereby exacerbating MPs-induced colonic inflammatory damage.^59,60^ Notably, BA metabolism is also intricately connected to gut microbiota, as secondary BAs are produced through the metabolism of primary BAs by gut microbiota. The interaction between gut microbiota and BA metabolism has been implicated in intestinal inflammation.^61^ Therefore, investigating the gut microbiota is essential. Our findings indicate that PS MPs treatment led to decreased gut microbiota diversity and altered composition. 16S rRNA sequencing revealed that the gut microbiota were primarily composed of bacteria, with over 90% belonging to Bacteroidota and Firmicutes. Following PS MPs treatment, the relative abundance of Bacteroidota decreased while Firmicutes increased. MPs exposure significantly suppressed gut microbiota with essential ecological functions, with the most pronounced reductions in the abundance of the probiotic (Lactobacillaceae family, decreased by 81.9%) and the potential probiotic (Akkermansiaceae family, decreased by 91.4%). plays a pivotal role in maintaining the intestinal barrier function by providing energy to colonic epithelial cells, enhancing mucin secretion to promote beneficial bacterial colonization, and inhibiting pathogen adhesion.^62^ Additionally, exhibits anti-inflammatory properties by regulating immune responses mediated by SCFAs, including the suppression of pro-inflammatory factors (e.g., TNF-α, IL-6) and the secretion of anti-inflammatory factors (e.g., IL-10).^63^ , a mucin-degrading bacterium residing in the mucus layer, specifically degrades mucins to maintain mucus layer homeostasis. It constitutes 3%–5% of the human gut microbiota and is considered one of the most promising next-generation probiotics, playing a significant role in maintaining the intestinal epithelial barrier.^64^ Supplementation with live or heat-inactivated can improve the intestinal barrier function, regulate intestinal inflammation, and positively impact host metabolism and immunity.^65^ The sharp decline in these two bacteria may synergistically exacerbate the intestinal toxicity of PS MPs. In addition to the disruption of and , PS MPs also reduced the relative abundance of Bacteroides, which has been shown to ameliorate intestinal inflammation or enhance barrier function in mice.^66^ PS MPs inhibit probiotics that maintain barrier function, reduce the abundance of anti-inflammatory bacterial genera, disrupt mucus layer homeostasis, weaken microbiota-mediated immune regulation, and ultimately increase intestinal permeability. This, in turn, facilitates the accumulation of pro-inflammatory factors, exacerbating the pathological progression of colitis.

Long-term exposure to PS MPs disrupts gut microbiota homeostasis, particularly reducing the abundance of beneficial bacteria. Microbiota imbalance not only affects the progression of colitis but also may lead to dysregulation of BA metabolism in the colon. After primary conjugated BAs enter the intestine with bile, approximately 95% are actively reabsorbed in the terminal ileum and returned to the liver via enterohepatic circulation, while the remaining 5% of unconjugated BAs enter the colon. Notably, in mice and rats, BAs are almost exclusively conjugated with taurine in the colon (e.g., TCA and TCDCA).^67^ The gut microbiota hydrolyzes these conjugated BAs into free BAs (e.g., CA, CDCA) through deconjugation, and some bacteria further convert them into more hydrophobic secondary BAs (e.g., DCA, LCA) via 7α-dehydroxylation, with the majority ultimately excreted in feces.^68^ Deconjugation of BAs prevents their accumulation in colonic epithelial cells, thereby reducing cytotoxicity.^69^ The key enzyme responsible for this deconjugation is bile salt hydrolase (BSH), which is produced by gut microbiota to hydrolyze conjugated BAs into free forms, mitigating their toxicity. Core gut microbiota that possess BSH include , , , and .^70^ In the present study, the abundance of was significantly reduced, while and within showed a significant negative correlation with conjugated BAs such as TCDCA. The decline in these bacteria may lead to reduced BSH activity, impairing the deconjugation of conjugated BAs and resulting in their accumulation in the colon. Significant increase in conjugated BAs may limit the substrates available for 7α-dehydroxylation, thereby reducing the production of secondary BAs such as LCA, which aligns with our BA metabolomics analysis. In summary, long-term exposure to PS MPs disrupts hepatic BA metabolism, enhancing BA synthesis and leading to excessive conjugated BAs entering the intestine. Concurrently, it negatively affects the gut microbiota, reducing the abundance of BSH-producing bacteria and impairing the hydrolysis of conjugated BAs. Consequently, the accumulation of conjugated BAs in the colon may induce colonic epithelial toxicity, further exacerbating PS MPs-induced colitis. However, the mechanisms through which BAs contribute to colonic epithelial toxicity in this model and whether they play a role in promoting PS MPs-induced colonic inflammatory injury remain unclear and require further investigation.

Building on previous findings, TCDCA, a conjugated BA, was selected as a representative compound for both in vitro and in vivo experiments. Studies have shown that TCDCA inhibits the proliferation of intestinal epithelial cells and induces apoptosis by activating the caspase system, independent of FXR. Additionally, TCDCA has been reported to cause diarrhea, liver injury, and even increase apoptosis in gastric cancer cells, thereby inhibiting cancer cell proliferation.^28^ Our results demonstrate that a specific concentration of TCDCA, in combination with PS MPs, significantly exacerbates cellular oxidative stress, leading to a substantial increase in the level of ROS in colonic epithelial cells. This excess ROS induces oxidative damage to mitochondria, activating mitochondrial apoptotic pathways, which ultimately results in the widespread death of colonic epithelial cells. Cellular and molecular biology experiments provided preliminary insights into the toxic mechanisms of TCDCA on colonic epithelial cells in vitro. To further investigate its role in vivo, a mouse model of PS MPs-induced colitis was used and mice were administered a specific concentration of TCDCA under inflammatory conditions. The results showed that the MP + TCDCA group exhibited significantly shortened colons, with H&E pathological staining revealing markedly aggravated colitis, including necrosis, and a substantial increase in inflammatory factors. These findings suggest that TCDCA significantly exacerbates PS MPs-induced colonic inflammatory injury in mice. Moreover, the interaction between gut microbiota and BAs appears to play a key role in the mechanism by which TCDCA aggravates PS MPs-induced colitis. An intriguing phenomenon occurred when broad-spectrum antibiotics were used to clear the gut microbiota in mice. Under these conditions, the colitis induced by PS MPs was significantly alleviated. This suggests that targeting the gut microbiota may offer a potential strategy to mitigate MPs-related intestinal damage, warranting further investigation in future studies.

However, this study has some limitations. First, only one particle size and polymer type of NPMs were used, limiting the generalizability of the findings. Future studies will include microplastics with more diverse materials and larger particle sizes to improve the environmental relevance. Second, we focused on a fixed dose of 10 mg/mL (1 mg/day), which did not cover environmentally relevant low-dose exposures or consider interspecies differences in microplastic toxicity. We will further refine the dose design and adopt organoid models to explore dose-dependent toxicity and improve clinical relevance. Finally, this study did not further elucidate the specific mechanisms by which microplastics affect BA metabolism in the liver and immune regulation. In addition, microbiota intervention experiments were lacking to verify their causal role in BA dysregulation. These mechanisms will be further investigated in future studies.

## 5 Conclusion

In conclusion, this study provides a comprehensive investigation into the mechanisms by which long-term oral exposure to 10 μm PS MPs induces colonic inflammation and injury in mice (Figure 11). Prolonged exposure to PS MPs disrupts the colonic redox system, elevates ROS levels, and triggers oxidative stress. Furthermore, PS MPs increase the Th17/Treg cell ratio and elevate pro-inflammatory cytokine levels, resulting in the disruption of intestinal immune homeostasis. Additionally, PS MPs reduce intestinal mucin secretion and decrease the expression of TJ proteins, compromising the mechanical barrier of the intestinal mucosa and impairing the intestinal barrier function. Simultaneously, PS MPs negatively impact liver function, leading to a significant increase in the level of TBA secretion. Multiomics analysis indicated that PS MPs induce BA metabolism dysregulation and gut microbiota imbalance, both of which are closely interrelated and mutually influential. Additionally, a specific concentration of TCDCA significantly exacerbates PS MPs-induced damage to the colonic epithelial cells. The mechanism involves increasing intracellular ROS levels, causing a significant decline in the mitochondrial membrane potential, which triggers mitochondria-mediated apoptosis and ultimately exacerbates colonic inflammation and injury, as evidenced by histopathological changes.

**Figure 11:**
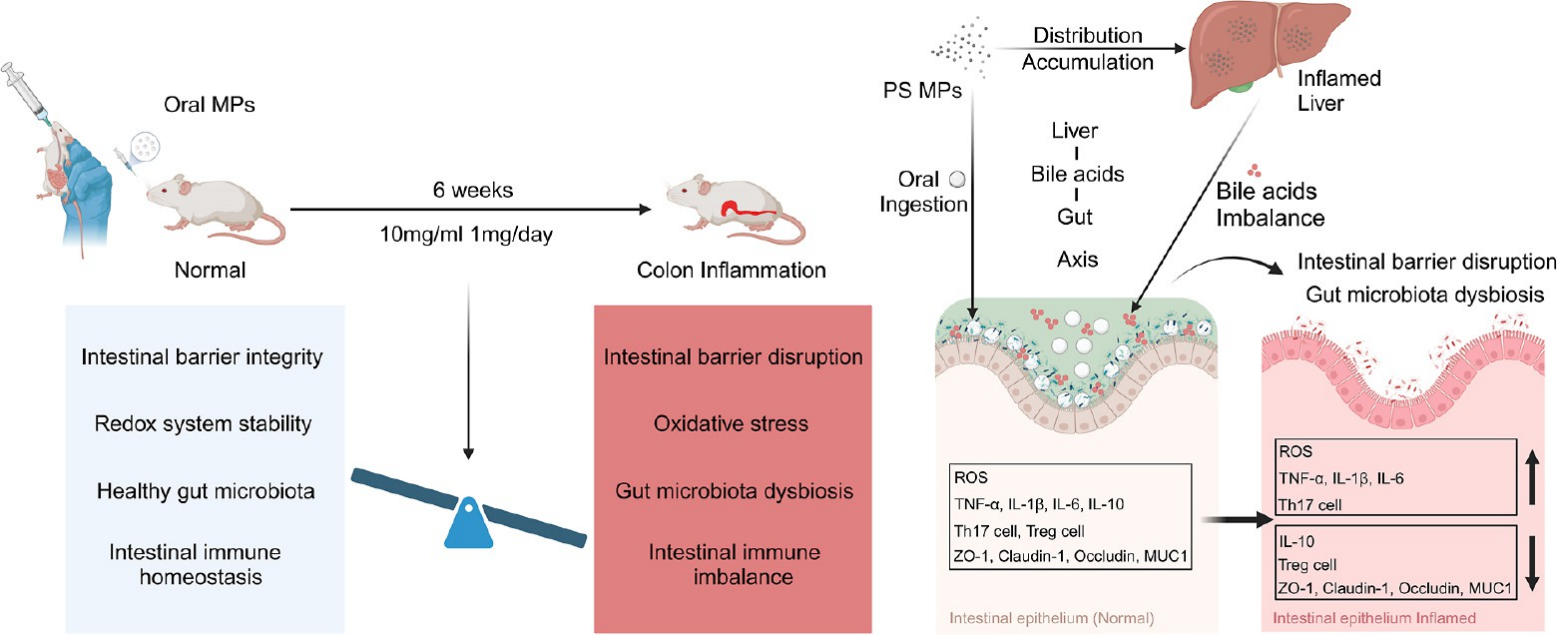
Schematic diagram of the basic mechanisms investigated in this study.

## Supplementary Material



## Notes

In vitro cellular uptake of PS MPs; long-term exposure to PS MPs: changes in mouse weight and distant organs, colonic GSSG/GSH expression levels, Th17/Treg flow cytometry analysis, BA metabolomics and fecal 16S rRNA; and in vitro and in vivo validation: CCK-8 and Western blotting data, comparison of mouse weight data, ELISA quantitative data of IL-10 (PDF)
