# Supplementary material for: Multiomics Reveals Nonphagocytosable Microplastics Induce Colon Inflammatory Injury via Bile Acid-Gut Microbiota Interactions and Barrier Dysfunction
Source: ACS Appl Mater Interfaces. 2025 Jul 19;17(31):44138–59. doi: 10.1021/acsami.5c07250 (PMC12332821; doi:10.1021/acsami.5c07250)
Supplement: Supplementary file 1 [file am5c07250_si_001.pdf]

Supporting Information

Multi-Omics Reveals Non-Phagocytosable Microplastics Induce Colon Inflammatory Injury *via* Bile Acid-Gut Microbiota Interactions and Barrier Dysfunction

Author Information

Junjie Chen[1#], Yixian Cheng[1#], Rui Fu[1#], Xinyu Chen[1#], Peng Zhang[1], Yixiao Lu[1], Bingsheng Liu[1], Peng Chen[2], JiahaoWang[2], Haikun Cao[3], Jinghua Gu[1], Haosong Chen[1], Zilong Jiang[4], Ting Li[1]*, Jiawei Zhang[1]*, Bo Chen[1]*, Guodong Cao[1]*

[1]Department of General Surgery, The First Affiliated Hospital of Anhui Medical University, Hefei, Anhui, China, 230022.

[2]Department of General Surgery, The Second Affiliated Hospital of Anhui Medical University, Hefei, Anhui, China, 230601.

[3]Department of Surgical Oncology, The First Affiliated Hospital of Bengbu Medical University, Bengbu, Anhui, China, 233004.

[4]Department of Medical Oncology, The First Affiliated Hospital of Anhui Medical University, Hefei, Anhui, China, 230022.

# These authors contributed equally to this work.

* Correspondence to Ting Li，E-mail: doctorliting@126.com; Jiawei Zhang, E-mail: zhangjiawei@ahmu.edu.cn; Bo Chen, E-mail: chenbo831116@163.com; Guodong Cao, E-mail: ayfycgd@163.com;

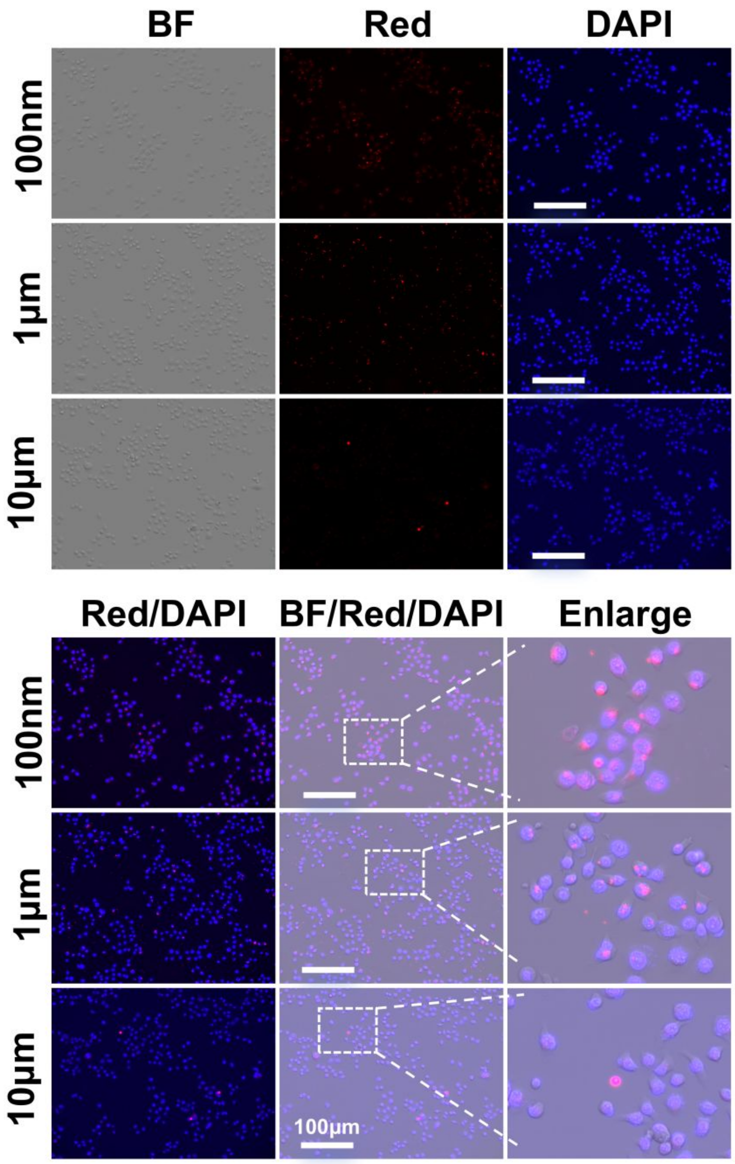

Figure S1. Cell uptake and *in vivo* distribution and accumulation of PS MPs. The cellular uptake of red fluorescent PS MPs with three different particle sizes co-incubated with NCM460 cells for 24 hours was examined. BF: bright field channel, Red: red fluorescence channel, DAPI: DAPI channel. BF/Red/DAPI: merged image. Enlarge: enlarged image. Scale bar: 100μm.

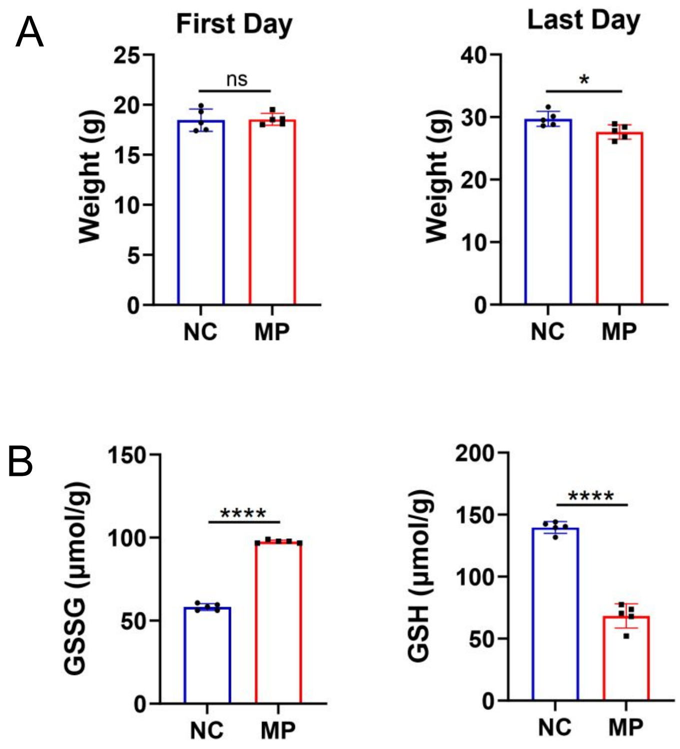

Figure S2. Long-term exposure to PS MPs results in colonic inflammation and oxidative stress. (A) Comparison of colonic weight between the NC group and the MP group on the first and last days during gavage with PS MPs. (B) Expression levels of oxidative stress markers GSSG, GSH in colonic tissues. *p < 0.05, **p < 0.01, ***p < 0.001, ****p < 0.0001, $n=5$.

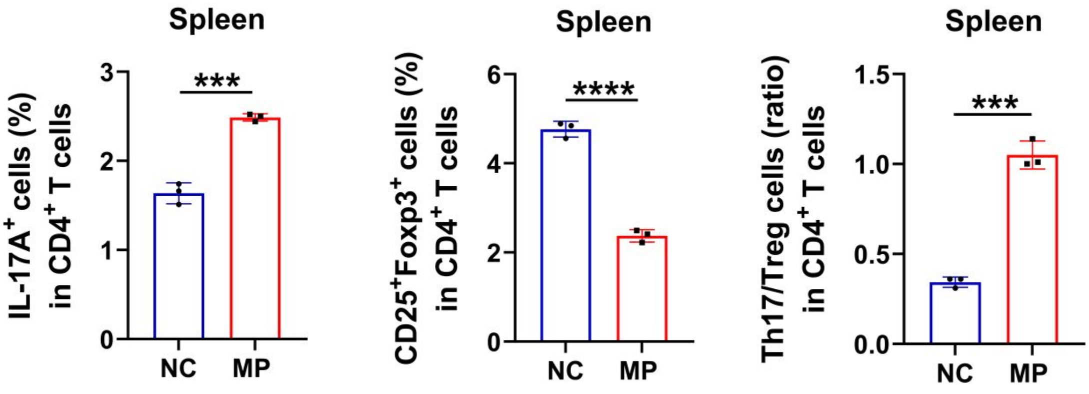

Figure S3. PS MPs disrupt the Th17/Treg balance and damage the intestinal mucosal barrier in mice. Flow cytometry analysis of the percentages of splenic CD4+ IL17A+ (Th17) and CD4+CD25+Foxp3+ (Treg) cells. Statistical analysis of the proportions of Th17 and Treg cells among splenic CD4+ T cells, along with the Th17/Treg ratio, is shown. ***$p < 0.001$, ****$p < 0.0001$, $n=3$.

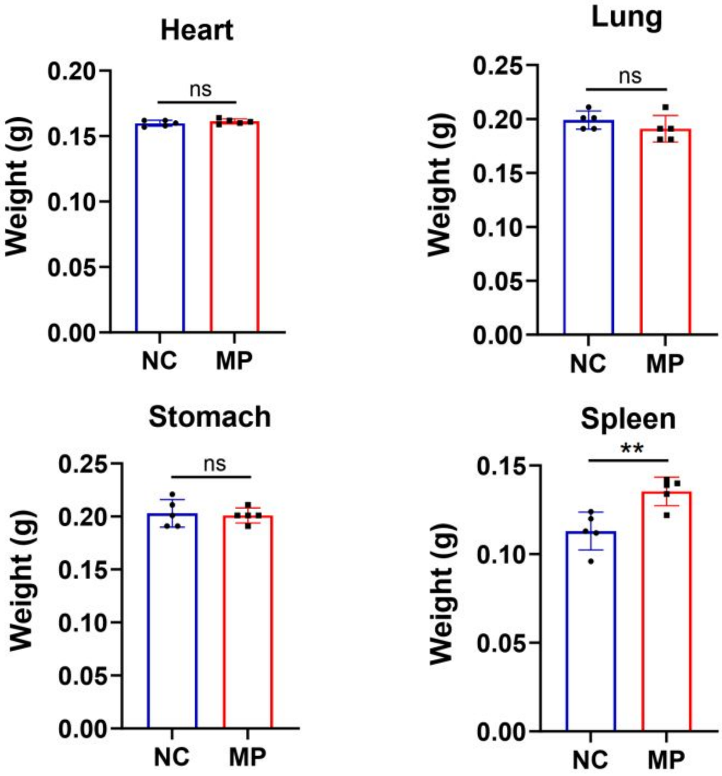

Figure S4. PS MPs induce liver damage and abnormal bile acid secretion. Statistical analysis of the weights of specimens from the heart, lungs, stomach and spleen between the two groups. **p < 0.01, n=5.

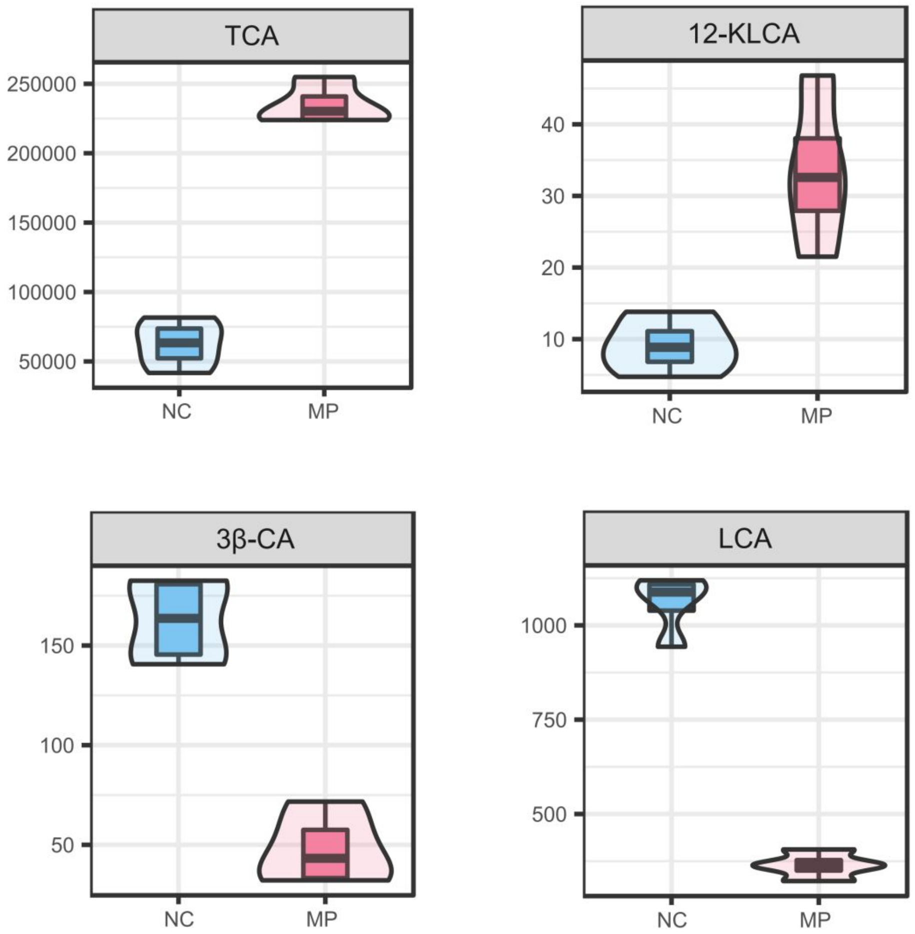

Figure S5. Fecal bile acid metabolomics indicates PS MPs-induced abnormal hepatic bile acid secretion. Violin plot of differential bile acids. *n=4*.

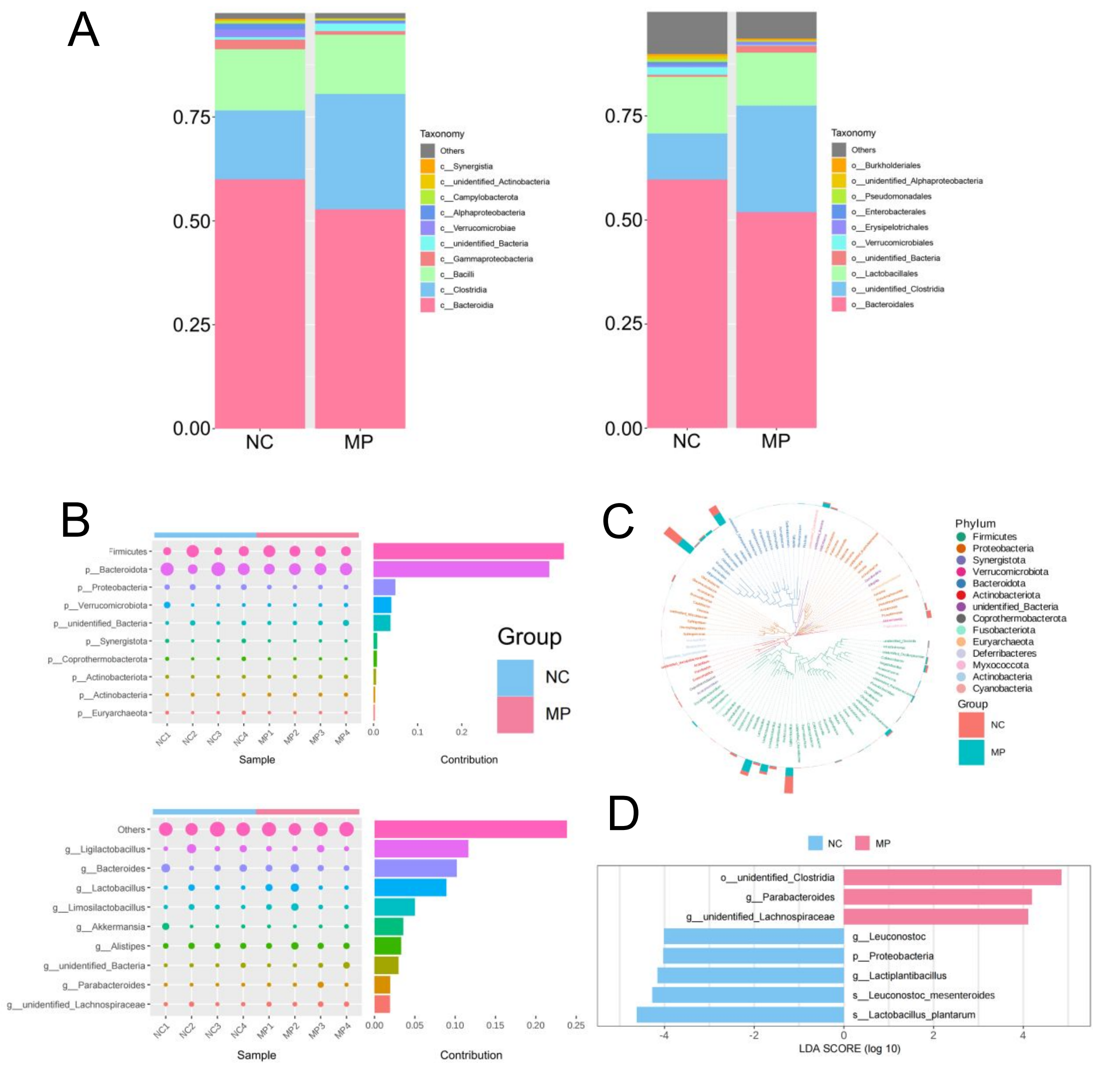

Figure S6. Alterations of the gut microbiota in the PS MPs group. (A) Composition of intestinal microbiota at the class and order levels. (B) Simper (Similarity Percentage) analysis of differential contributions. Bubble size represents the relative abundance of the species, and contribution indicates the species' contribution to the differences between the two groups. (C) Representative sequences and abundance distribution of intestinal microbiota at the genus level. (D) Linear discriminant analysis effect size (LEfSe) method used to identify key taxonomic units in different groups. **p* < 0.05, ***p* < 0.01, *n=4*.

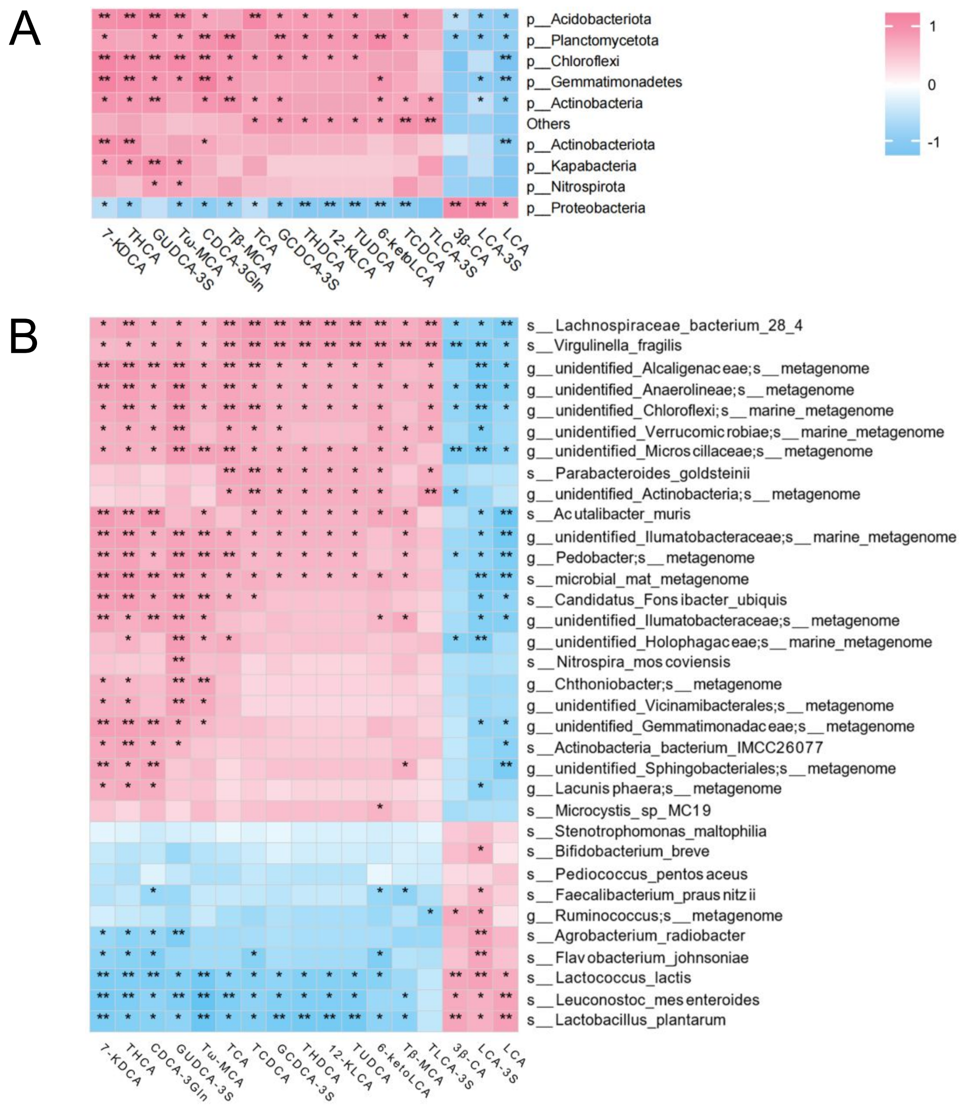

Figure S7. Alterations of the gut microbiota in the PS MPs group. (A) Spearman correlation clustering heatmap at the phylum level and (B) at the species level showing the differential microbial communities in mice after PS MPs treatment and their correlation with differential bile acids. Red: positive correlation; blue: negative correlation. *$p < 0.05$, **$p < 0.01$, $n=4$.

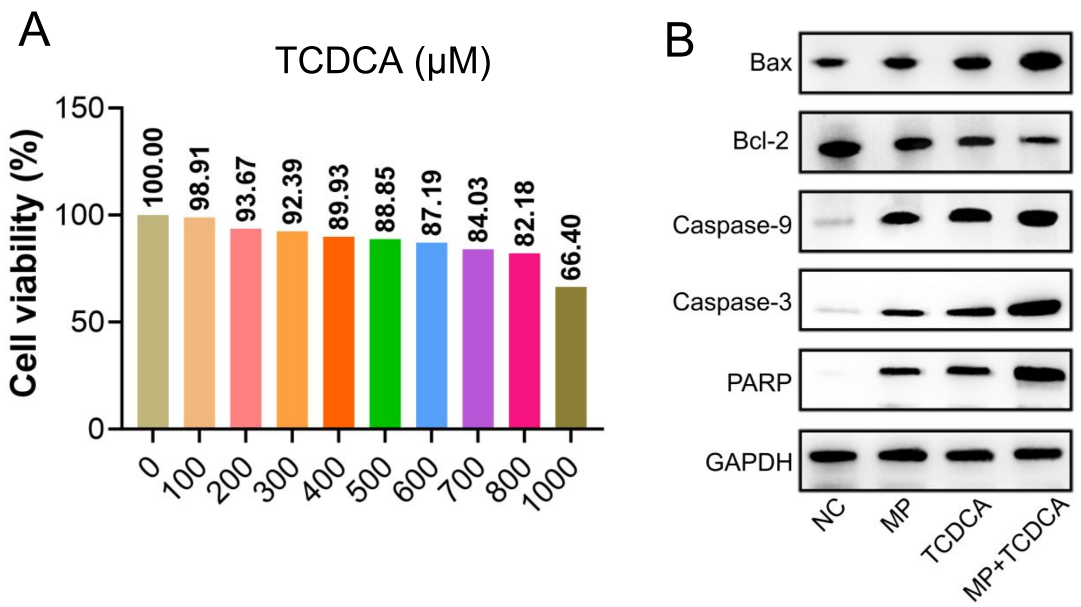

Figure S8. Bile acid TCDCA exacerbates PS MPs-induced apoptosis in colon epithelial cells. (A) CCK-8 cell proliferation and toxicity assay. (B) Western blotting detection of mitochondrial apoptosis-related proteins. *n=3*.

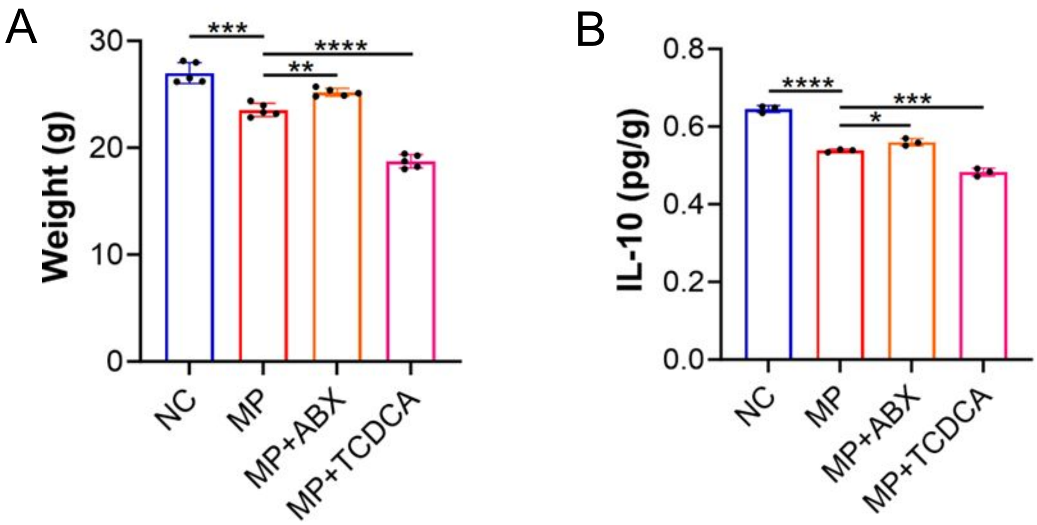

Figure S9. Bile acid TCDCA exacerbates PS MPs-induced colitis and intestinal injury in mice. (A) Statistical analysis and comparison of the weight in different groups of mice. *n=5*. (B) ELISA detection of expression levels of inflammatory factors IL-10 in mouse colon. *n=3*. $*p < 0.05$, $**p < 0.01$, $***p < 0.001$, $****p < 0.0001$.
